# NBCe1-B/C-knockout mice exhibit an impaired respiratory response and an enhanced renal response to metabolic acidosis

**DOI:** 10.3389/fphys.2023.1201034

**Published:** 2023-06-19

**Authors:** Clayton T. Brady, Aniko Marshall, Chen Zhang, Mark D. Parker

**Affiliations:** ^1^ Jacobs School of Medicine and Biomedical Sciences, Department of Physiology and Biophysics, The State University of New York: The University at Buffalo, Buffalo, NY, United States; ^2^ Department of Biological Sciences, The State University of New York: The University at Buffalo, Buffalo, NY, United States; ^3^ Jacobs School of Medicine and Biomedical Sciences, Department of Ophthalmology, The State University of New York: The University at Buffalo, Buffalo, NY, United States

**Keywords:** NBCe1, acid-base, acidosis, bicarbonate, transport, kidney, brainstem

## Abstract

The sodium-bicarbonate cotransporter (NBCe1) has three primary variants: NBCe1-A, -B and -C. NBCe1-A is expressed in renal proximal tubules in the cortical labyrinth, where it is essential for reclaiming filtered bicarbonate, such that NBCe1-A knockout mice are congenitally acidemic. NBCe1-B and -C variants are expressed in chemosensitive regions of the brainstem, while NBCe1-B is also expressed in renal proximal tubules located in the outer medulla. Although mice lacking NBCe1-B/C (KO_b/c_) exhibit a normal plasma pH at baseline, the distribution of NBCe1-B/C indicates that these variants could play a role in both the rapid respiratory and slower renal responses to metabolic acidosis (MAc). Therefore, in this study we used an integrative physiologic approach to investigate the response of KO_b/c_ mice to MAc. By means of unanesthetized whole-body plethysmography and blood-gas analysis, we demonstrate that the respiratory response to MAc (increase in minute volume, decrease in pCO_2_) is impaired in KO_b/c_ mice leading to a greater severity of acidemia after 1 day of MAc. Despite this respiratory impairment, the recovery of plasma pH after 3-days of MAc remained intact in KO_b/c_ mice. Using data gathered from mice housed in metabolic cages we demonstrate a greater elevation of renal ammonium excretion and greater downregulation of the ammonia recycling enzyme glutamine synthetase in KO_b/c_ mice on day 2 of MAc, consistent with greater renal acid-excretion. We conclude that KO_b/c_ mice are ultimately able to defend plasma pH during MAc, but that the integrated response is disturbed such that the burden of work shifts from the respiratory system to the kidneys, delaying the recovery of pH.

## 1 Introduction

Regulation of pH at the level of the cell, organ, and whole organism is an essential component of health. Acidemia has been implicated in osteopenia and osteoporosis (Weger et al., 2000), decreased insulin release and sensitivity (Farwell and Taylor, 2008), vascular-endothelial dysfunction (Wesson et al., 2011), progression of chronic kidney disease to end-stage renal disease (Shah et al., 2009; Phisitkul et al., 2010), cardiac arrhythmias (Orchard and Cingolani, 1994), and heart failure (Urso et al., 2015). The maintenance of a normal plasma pH (7.35–7.45) requires contributions from multiple organ-systems, creating a complex feedback network. In particular, the lungs and kidneys function together to maintain the ratio of the partial pressure of carbon dioxide (pCO_2_) to the plasma bicarbonate concentration ([HCO_3_^−^]), as governed by the classic Henderson-Hasselbalch equation ( $pH=6.1+\log\;\frac{\left[HCO_{3}^{-}\right]}{0.03*pCO_{2}}$ ).

During metabolic acidosis (MAc [HCO_3_^−^] <22 mEq/L), chemoreceptors in the brain and periphery (i.e., aortic and carotid bodies) stimulate increases in lung ventilation in order to reduce pCO_2_ and mitigate the fall in plasma pH (O’Regan and Majcherczyk, 1982; Schuitmaker et al., 1987). The kidneys respond to MAc primarily through the metabolic process ammoniagenesis, which results in *de novo* production of equimolar HCO_3_^−^ and ammonium (NH_4_^+^); HCO_3_^−^ is transported into circulation and NH_4_^+^ is excreted in the urine (Weiner and Verlander, 2019). Upregulation of ammoniagenesis requires the coordinated expression changes of enzymes involved in the metabolism of glutamine to NH_4_^+^ and HCO_3_^−^. Two of these enzymes include phosphoenolpyruvate carboxykinase (PEPCK), and glutamine synthetase (GS). PEPCK specifically catalyzes the formation of HCO_3_^−^ as part of the gluconeogenesis pathway, which leads to increased net HCO_3_^−^ production; thus MAc normally stimulates increased PEPCK expression (Curthoys and Gstraunthaler, 2001). On the other hand, glutamine synthetase (GS) is involved with the recycling of NH_4_^+^ by catalyzing the addition of NH_4_^+^ to glutamate forming glutamine, and thus MAc normally decreases GS expression in order to increase net NH_4_^+^ excretion (Conjard et al., 2003). While both distal and proximal tubule (PT) segments participate in ammoniagenesis, only PT ammoniagenesis contributes to the overall increase in NH_4_^+^ excretion seen during MAc (Good and Burg, 1984).

Recent work has advanced our understanding of the molecular mechanisms underlying these organ responses. Of increasing interest is the role of the Na^+^-HCO_3_^−^ co-transporter (NBCe1), encoded by the *SLC4A4* gene. Mutations in *SLC4A4* cause proximal renal tubular acidosis: HCO_3_^−^ wasting acidemia with a variety of extra-renal sequelae such as loss of vision, growth abnormalities, and intellectual disability (Igarashi et al., 1999; Salerno et al., 2019). NBCe1 has three primary protein variants (NBCe1-A, -B, and -C), each with a different expression pattern through the body (Brady et al., 2020). In the kidneys, NBCe1-A is exclusively expressed in PTs of the cortical labyrinth where it plays an essential role in both the reabsorption of filtered HCO_3_^−^ (Burnham et al., 1997; Romero et al., 1997; 1998) and the ammoniagenic response (Lee et al., 2018). Hence, NBCe1-A-knockout (KO) mice are spontaneously acidemic, as is a proband with an NBCe1-A-specific nonsense mutation (Igarashi et al., 2001; Lee et al., 2018). NBCe1-B is more widely expressed, and differs from NBCe1-A in that an 85 amino acid auto-inhibitory sequence replaces a 41 amino acid auto-stimulatory sequence (McAlear et al., 2006). In the kidneys, NBCe1-B is expressed in PTs of the outer segment of the outer medulla (OSOM) where NBCe1-A is absent (Fang et al., 2018). Moreover, NBCe1-B is transcribed from an acid-sensitive promoter, which suggests renal NBCe1-B may have a role in the kidneys response to acidosis (Snead et al., 2011; Fang et al., 2018). However, in contrast to spontaneously acidemic NBCe1-A-KO mice, NBCe1-B/C-KO (KO_b/c_) mice exhibit normal blood pH (Salerno et al., 2019). NBCe1-C is largely identical to NBCe1-B except that a 46 amino acid carboxy-terminal appendage is replaced by a 61 amino acid sequence of unknown physiological consequence (Bevensee et al., 2000). Both NBCe1-B and NBCe1-C are expressed in neurons (predominantly NBCe1-B) and astrocytes (predominantly NBCe1-C) in the brain; although this expression pattern is reversed in cultured cells and may even be species dependent (Majumdar et al., 2008; Virreira et al., 2019). Therefore, in the context of the brain we do not distinguish between–B and–C variants, and refer to them as ‘NBCe1-B/C’ in this text.

There are eight central chemoreceptor sites, located within the brainstem, cerebellum, midbrain, and hypothalamus [reviewed in (Nattie and Li, 2012)]. Relevant to this study, NBCe1-B/C was demonstrated to contribute to the cellular mechanism underlying the chemosensitivity of astrocytes located on the ventral surface of the brainstem medulla (Turovsky et al., 2016), which are adjacent to neuronal cell bodies that comprise the retrotrapezoid nucleus (RTN) (Erlichman et al., 2004; Gourine et al., 2010; Kasymov et al., 2013; Sheikhbahaei et al., 2018). The RTN is considered the prototypical respiratory chemoreceptor for detecting changes in pH/pCO_2_ and mediating changes in ventilation (Guyenet et al., 2019). While not considered a primary contributor to the generation of baseline respiratory patterns, the RTN rather modifies breathing in response to changes in pH (such as MAc) and/or pCO_2_ (Burke et al., 2015; Souza et al., 2018). The molecular mechanism underlying RTN chemosensitivity is still controversial; however, one hypothesis is centered on an NBCe1-B/C-mediated mechanism in which chemosensitive astrocytes respond to increased acidity/pCO_2_ by releasing ATP, which triggers action potentials in adjacent RTN neurons resulting in increased ventilation (Gourine et al., 2010; Turovsky et al., 2016). Nevertheless, evidence that loss of NBCe1-B/C attenuates central chemoreception *in vivo* is lacking.

The fact that NBCe1-B/C, appears positioned to contribute to both renal (NBCe1-B) and respiratory (NBCe1-B/C) responses to MAc, suggests that NBCe1-B could be a fundamental component of the cellular machinery underlying the integrated physiologic response to acid-base disturbance. Thus, the overarching goal of this study was to establish the role of NBCe1-B/C in control of whole-body acid-base balance. First, we validate a novel, commercially available, NBCe1-B/C specific antibody, which is used throughout the study in the context of both the brain and the kidney. Second, we describe the abundance responses of both NBCe1-A and NBCe1-B in kidney during MAc. Third, we describe the effect of NBCe1-B/C loss on the respiratory and renal responses during MAc using the KO_b/c_ mouse model (Salerno et al., 2019). Lastly, we examine the abundance response during MAc of PEPCK and GS in PTs of WT and KO_b/c_ mice.

## 2 Materials and methods

### 2.1 Ethical statement

All procedures involving animals were approved by and performed in accordance with the rules and recommendations of the Institutional Animal Care and Use Committee of the University at Buffalo.

### 2.2 Mice

The generation and genotyping of the KO_b/c_ mouse on a C57BL/6J background have been previously reported (Salerno et al., 2019). For this study, heterozygous parents were produced by backcrossing heterozygous mice with verified C57BL/6J wild-type mice (Jackson Laboratory, Bar Harbor, ME). Heterozygous progeny (F6 to F17 generation, making them at least 99% genetically identical) were crossed to produce experimental WT and KO_b/c_ mice for this study. As previously reported, KO_b/c_ mice exhibit increased mortality [50% at 5 weeks (Salerno et al., 2019)] so we were constrained to working with mice between 4–5 weeks of age in order to maximize the likelihood of survival during study, while still using mice past the age of full structural and functional kidney development (Satlin et al., 2003; Seely, 2017).

### 2.3 Metabolic studies and induction of metabolic acidosis

WT and KO_b/c_ mice between 4-5-weeks old were housed in metabolic chambers (Tecniplast, 3600M021) and given standard powered rodent chow (Teklad 8604). After a 1-day acclimation period with plain tap water, experimental groups were subjected to 1–3 days (duration depending on the experiment) of control or MAc-challenged conditions. MAc was induced by adding 0.28 M ammonium chloride (NH_4_Cl) + 0.5% sucrose (for palatability) in drinking water (tap water). Control groups were given drinking water containing only 0.5% sucrose for the same 1–3-day duration. NH_4_Cl administration is a common method for induction of MAc and enhancement of ammoniagenesis in both humans and animals (Rector et al., 1955; Amlal et al., 2004; Alam et al., 2020). All mice had free access to powdered standard rodent chow for the duration of the experiment. During the acclimation period, body weight, food intake, and fluid intake were monitored. During the experimental period, urine and fecal excretion were also monitored, and urine was collected each day under mineral oil (to prevent evaporation). Urine samples were spun at 10,000 RCF for 5 min to remove any solid particles and frozen at −80°C until urinalysis. Since repeated measures of metabolic and urinary parameters were possible in mice subjected to experiments of 2-, and 3-day durations before sacrifice for plasma electrolyte analysis, this led to a larger sample size for these parameters for day 1, compared to day 2 or day 3. Sample sizes for mice kept under 1, 2, or 3 days of control or MAc-challenged conditions are shown in Table 1, which relates to the data presented in Table 2 and Figure 7.

**TABLE 1:** Sample size for metabolic cage experiments.

Group	Day 1	Day 2	Day 3
Control WT	n = 16M/14F	n = 11M/9F	n = 8M/6F
Control KO_b/c_	n = 15M/11F	n = 11M/7F	n = 7M/4F
MAc WT	n = 17M/19F	n = 11M/13F	n = 6M/9F
MAc KO_b/c_	n = 16M/13F	n = 11M/8F	n = 6M/4F
Sample sizes given as n = male/female and relates to data presented in Table 2 and Figure 7			

**TABLE 2:** Metabolic and electrolyte status of WT and KO_b/c_ mice over 1–3 days of control or MAc conditions.

Parameter	WT	KO_b/c_	*p*-value
Control conditions			
Body weight (g)	15.5 ± 0.2 (64)	13.9 ± 0.3 (55)	<0.001*
Daily food intake (g/day)	4.2 ± 0.1 (64)	3.7 ± 0.1 (55)	<0.001*
Daily food intake (g/day/body weight)	0.270 ± 0.004 (64)	0.271 ± 0.004 (55)	0.643
Daily fluid intake - 0.5% sucrose (mL/day)	6.1 ± 0.1 (64)	5.6 ± 0.1 (55)	0.022*
Daily fluid intake - 0.5% sucrose (mL/day/body weight)	0.39 ± 0.01 (64)	0.41 ± 0.01 (55)	0.229
Plasma [Na^+^], mEq/L	145.3 ± 0.5 (27)	144.0 ± 0.3 (24)	0.006*
Plasma [Cl^−^], mEq/L	109.9 ± 0.6 (26)	106.8 ± 0.4 (24)	<0.001*
BUN, mg/dL	31 ± 1 (27)	24 ± 1 (23)	<0.001*
MAc conditions			
Body weight (g)	14.6 ± 0.2 (75)	13.3 ± 0.3 (58)	0.003*
Daily food intake (g/day)	3.2 ± 0.1 (75)	2.9 ± 0.1 (58)	<0.001*
Daily food intake (g/day/body weight)	0.223 ± 0.004 (75)	0.219 ± 0.003 (58)	0.081
Daily fluid intake - 0.28M NH_4_Cl + 0.5% sucrose (mL/day)	5.1 ± 0.1 (75)	4.9 ± 0.3 (58)	0.083
Daily fluid intake - 0.28M NH_4_Cl + 0.5% sucrose (mL/day/body weight)	0.35 ± 0.01 (75)	0.37 ± 0.01 (58)	0.965
Plasma [Na^+^], mEq/L	149.1 ± 0.5 (32)	148.7 ± 0.3 (26)	0.657
Plasma [Cl^−^], mEq/L	120.7 ± 0.7 (32)	117.5 ± 1.2 (25)	<0.001*
BUN, mg/dL	40 ± 1 (32)	35 ± 2 (26)	0.001*

Values represent mean ± SEM, from all experiments where animals were kept under 1, 2, or 3-days of control or metabolic acidosis (MAc) conditions. Numbers in parentheses are the numbers of animals in each group. See Table 1 for per-day sample sizes. *p*-values calculated by 2-way ANOVA.

### 2.4 Plethysmography

The breathing activity of unrestrained mice was measured without anesthesia as described previously (Morse et al., 2012). Individual animals were kept in a plexiglass plethysmography chamber connected to an empty reference chamber for buffering changes in atmospheric pressure (PLY4213, Buxco Research Systems, Wilmington, NC). The plethysmograph was connected to a Rodent Bias Flow Supply (BFL0250) to draw expired CO_2_ out of the chamber and provide a constant flow of room air at a flow rate of 2.5 L/min. Signals were collected with a barometric pressure sensor attached to a port on the reference chamber. The sensor was connected to a MAX 1500 preamplifier, and signals were visualized and collected using BioSystem XA software. The chamber and software were calibrated for mice at the beginning of each experiment using an injection of 1 mL of air through the base port of the chamber. To limit interference, exclusion parameters were set as follows: Max expiration time–0.3 s, Min Tidal volume–0.02 mL, Min Inspiration time–0.03 s. Recordings were taken once mice settled into a period of quiet breathing, after a minimum 30-min acclimation period. Each recording consisted of 15 min, during which tidal volume, frequency, and minute volume were averaged over 5 s intervals. Periods of activity or sniffing were noted and excluded from analysis. Tidal volume and minute volume were normalized to body-weight. Baseline parameters were gathered by averaging data from 2 consecutive days of mice housed in unchallenged conditions, prior to each mouse being given a 3-day MAc challenge.

### 2.5 Blood gas measurements

To obtain repeated daily blood pCO_2_ and pH measurements in mice without anesthesia, mice were restrained using 50 mL conical tube, modified by cutting a breathing hole at the conical end and a hole for tail access through the screw cap. After allowing 5 min for the mice to acclimate, an incision was made in the lateral tail vein using a 18G needle. 6–10 µL of blood was drawn from the incision using a P10 pipette and immediately transferred to a 1.5 mL Eppendorf containing 25 µL of prewarmed (37°C) mineral oil to prevent degassing of the blood sample. pCO_2_ was immediately measured using a micro-carbon dioxide electrode (MI-720; Microelectrodes Inc, Bedford NH), that had been calibrated in 1L CO_2_/HCO_3_^−^-containing solutions gassed to 3% (22.8 mmHg), 5% (38 mmHg), or 8% (60.8 mmHg) CO_2_ concentrations at pH 7.4 and maintained at 37°C (in mM: 5 HEPES, 10 glucose, 5 KCl, 0.8 MgCl_2_, 1.35 CaCl_2_, [127, 119, or 106] NaCl, [13, 21, or 34] NaHCO_3_; the varying NaCl and NaHCO_3_ concentrations represent those used for the 3%, 5%, and 8% CO_2_ solutions, respectively). Following measurement of pCO_2_, pH was immediately measured using a micro-pH electrode (Orion 9810BN, Thermo Fisher Scientific). Calculation of [HCO_3_^−^] was derived from the Henderson-Hasselbach equation ( $pH=6.1+\log\;\frac{\left[HCO_{3}^{-}\right]}{0.03*pCO_{2}}$ ). Specifically [HCO_3_^−^] = 0.03 x (pCO_2_ in mmHg) x 10^(pH–6.125)^.

### 2.6 Blood electrolyte measurements

Mice were anesthetized with isoflurane (5%, inhaled) and blood drawn by cardiac puncture was immediately transferred into a blood metabolite test card and analyzed using an Epoc reader according to the manufacturer’s instructions (both Siemens Medical Solutions United States Inc., Malvern, PA).

### 2.7 Urinalysis

24-h urine collections obtained from mice housed in metabolic cages for 1–3 days (see ‘Metabolic Studies’ above) were used to assess daily NH_4_^+^ excretion, daily TA excretion, and daily urine pH. Since mice were sacrificed after each experimental duration for electrolyte analysis, there are larger sample sizes for day 1 than for day 2 or day 3 within the urinalysis data set (Figure 7). Urine NH_4_^+^ concentrations were assessed using a commercially available kit (AA0100, Sigma-Aldrich, St. Louis, MI) that was modified for use in 96-well plates, with samples diluted 1:100 and ran in triplicate. Daily NH_4_^+^ excretion rates were calculated using 24-h urine NH_4_^+^ concentrations and volumes. Titratable acid (TA) content was measured as described previously (Chan, 1972; Lee et al., 2009). Briefly, 25 µL of 0.1 M HCl was added to 25 µL of urine, boiled for 2 min, and let cooled for 1 min. The volume of 0.4 M NaOH needed to bring the sample pH to 7.4 was quantified. Samples of distilled (DI) water were run in parallel. The total number of moles needed to bring samples to pH 7.4, less the moles needed to bring the DI water to pH 7.4, was multiplied by 24-h urine volumes to yield daily TA excretion. Urine pH was measured using a micro-pH electrode (Orion 9810BN, Thermo Fisher Scientific).

### 2.8 Antibodies

The Total-NBCe1 antibody (affinity for all NBCe1 protein variants) was purchased from Elabscience Biotechnology, Inc. (E-AB-14348, Houston TX; raised in rabbit), and has been previously validated (Salerno et al., 2019). The Total-NBCe1 antibody was used at a 1:1,000 concentration for Western blot. The NBCe1-A specific antibody generously gifted by Dr. Michael Romero (Mayo Clinic, Rochester MN; raised in chicken) has been previously validated (Lee et al., 2018), and was used at a 1:1,000 concentration for Western blot. The NBCe1-B/-C antibody was purchased from Santa Cruz Biotechnology, Inc. (sc-515543, Dallas TX; raised in mouse), and was used at a 1:1,000 concentration for Western blot and 1:100 dilution for immunohistochemistry (IHC). The NBCe1-B/C antibody was raised against an epitope that corresponds to the first 41 amino acids of the unique amino-terminal sequence of NBCe1-B/C (MEDEAVLDRGASFLKHVCDEEEVEGHHTIYIGVHVPKSYRR). The Phox2B antibody was purchased from Thermo Fisher (PA5-115754; raised in rabbit), and was used at a 1:500 concentration for IHC. The glutamine synthetase (GS) antibody was purchased from Abcam (ab73593; raised in rabbit), and was used at a 1:20,000 concentration for IHC. The phosphoenolpyruvate carboxykinase (PEPCK) antibody was purchased from Cayman Chemicals (10004943; raised in rabbit), and was used at a 1:10,000 dilution for IHC.

The secondary HRP-conjugated anti-chicken immunoglobulin antibody was purchased from Thermo Fischer Scientific (A16054; raised in goat), and used at a 1:2,000 dilution for Western blot and 1:1,000 dilution for IHC. The secondary HRP-conjugated anti-mouse immunoglobulin antibody was purchased from MP Biomedicals (55563, Solon OH; raised in goat), and used at a 1:2,000 dilution for both Western blot and 1:1,000 dilution for IHC. The secondary HRP-conjugated anti-rabbit immunoglobulin antibody was purchased from MP Biomedicals (55685, Solon OH; raised in goat), and used at a 1:1,000 dilution for IHC.

### 2.9 Oocyte preparation

Oocytes were extracted from female *Xenopus laevis* frogs (*Xenopus* Express, Brooksville, FL) as described elsewhere (Musa-Aziz et al., 2010). In brief, ovaries of anesthetized frogs (0.2% tricaine) were surgically removed and washed in Ca^2+^-free “NRS” buffer (in mM: 82 NaCl, 2 KCl, 20 MgCl_2_, 5 HEPES, adjusted to pH 7.45 with NaOH). Frogs were then euthanized via exsanguination. Oocytes were extracted from ovaries by digestion in NRS buffer plus 2 mg/mL type-IA collagenase (C2674: Sigma-Aldrich, St. Louis, MO) for 15–35 min, and washed in NRS buffer, followed by ND96 solution, and finally with OR3 medium. OR3 medium contains 14 g/L of Leibovitz’s L-15 medium (10–045-CV, Thermo Fisher Scientific), 100 U/mL penicillin, 100 μg/mL streptomycin, 5 mM HEPES, and adjusted to pH 7.5 using NaOH. The osmolality of OR3, measured using a Vapro vapor pressure osmometer (Wescor, Logan, UT), was adjusted to 195 ± 5 mOsmol/kg osmolality with H_2_O.

The construction of the plasmids for expression of WT human NBCe1-A or NBCe1-B in *Xenopus* oocytes has been described previously (Lee et al., 2011; Myers et al., 2016b). Each construct included a carboxy-terminal enhanced green fluorescent protein (EGFP) tag (NBCe1-A-EGFP.pGH19 and NBCe1-B-EGFP.pGH19) used to confirm expression in oocytes before homogenization. Plasmids were transformed into *E. coli* and cultured overnight. After isolation, plasmids were linearized using NotI, followed by purification using the MinElute PCR Purification Kit (28004, Qiagen). Linearized DNA was transcribed to capped cRNA using the T7 mMessage mMachine Transcription kit (Invitrogen, Carlsbad, CA). cRNA was further purified with the RNeasy MinElute Cleanup kit (74204, Qiagen) before quantification using a Nanodrop 2000 (Thermo Fisher Scientific). A 25 nL bolus of cRNA (or water for negative control) was injected into oocytes using a Nanoject III programmable nanoliter injector (Drummond Scientific Co., Broomall, PA).

### 2.10 Western blotting

*Xenopus* oocytes: Oocytes were prepared as previously described (Myers et al., 2016a). In brief, cells were incubated for 3 days in OR3 medium to allow for protein expression and cells were homogenized in TBS buffer containing 1% Triton X-100 and Complete Protease Inhibitor Cocktail. Yolk platelets and other insoluble components were pelleted by low-speed centrifugation: 850x RCF for 5 min. The supernatant was mixed with gel-loading buffer and the equivalent of ¼ an oocyte was loaded into each well for Western blotting.

Mouse kidneys: Excised kidneys were placed into of ice-cold homogenization buffer (HB; in mM: 100 NaCl, 25 HEPES, 250 sucrose, pH 7.4) plus cOmplete Protease Inhibitor Cocktail (Pierce A32963, Thermo Fisher Scientific). For separation of cortical and medullary protein samples, the cortex and medulla of freshly excised kidneys were separated under a dissecting microscope, guided by the contrast in color of the two zones, prior to homogenization. Whole kidney, cortical, or medullary membrane protein homogenates were prepared from dissected tissue using fractional centrifugation techniques. Briefly, tissue was homogenized with a handheld homogenizer (D1000, Benchmark Scientific, Edison, NJ), resuspended in 5 mL of HB, and spun at 1075 RCF for 15 min with the resulting supernatant retained. Membrane fragments were precipitated from the supernatant by ultracenxtrifugation using a Beckman Optima L-70 Ultracentrifuge (Beckman Coulter Inc., Indianapolis, IN) at 204,300x RCF for 45 min. The resulting pellet was resuspended in 300 µL HB. Homogenate protein concentrations were determined using the BCA colorimetric assay (Pierce 23227, Thermo Fisher Scientific), modified for microplate conditions. Immediately before gel loading, samples were pre-treated with a 0.1 M dithiothreitol (DTT) solution and denatured with LDS (Invitrogen NP0007, Thermo Fisher Scientific). DTT treatment is necessary to remove interfering endogenous mouse immunoglobulin for NBCe1-B probed kidney blots since the secondary antibody recognizes mouse immunoglobulin which, when intact, interferes with the measurement of NBCe1-B abundance (Brady; unpublished observations). DTT reduces the disulfide bonds of intact immunoglobulin resulting in lower molecular weight subunits that do not interfere with the NBCe1 band (Ahmad-Zadeh et al., 1971). DTT treatment is also known to dissociate NBCe1 dimers (Kao et al., 2008), resulting in only bands representing NBCe1 monomer abundance. Protein (10 µg/lane, unless specified) was resolved on a 3%–8% Tris-Acetate gel (Invitrogen EA0378BOX, Thermo Fisher Scientific) and transferred onto a PVDF membrane (Invitrogen LC 2005, Thermo Fisher Scientific). For studies of NBCe1-A and NBCe1-B abundance, age- and sex-matched littermates were paired between control and experimental groups, and each sample was run in duplicate on the same gel as its pair. To confirm even protein loading, prior to antibody application, each blot was treated with the reversible Memcode total-protein stain and blots with clearly unequal loading were discarded (Pierce 24585, Thermo Fisher Scientific). Additionally, Memcode stained blots were imaged using the visible light setting of a myECL imager (Thermo Fisher Scientific), and used to normalize differences in protein between lanes (Janes, 2015). After the total protein was imaged and the stain was erased, the PVDF membrane was incubated overnight in Tris-buffered saline (TBS; in mM: 10 HCl, 150 NaCl) containing 0.1% Tween-20 (0.1% TBS-T) and 4% milk powder at 4°C. The next day, the membrane was probed with primary antibody diluted in 2% milk powder prepared in 0.1% TBS-T, followed by an HRP-conjugated secondary antibody diluted similarly. Immunoreactive protein bands were visualized and imaged using ECL2 reagent (Pierce 32106, Thermo Fisher Scientific) and the chemiluminescent signal was imaged using the myECL imager after confirming the absence of pixel saturation. Densitometry was performed using FIJI software on both total-protein stained and antibody probed blots (Schindelin et al., 2012; Janes, 2015). The images of the total-protein stained blots were background subtracted using the FIJI rolling ball subtraction algorithm with a 100-pixel radius. Total-protein was quantified using densitometry of the entire lane profile plotted from rectangles drawn around each lane of the blot. Individual band densitometry results from the antibody-probed blots were normalized to their respective total-protein results and these normalized ratios were subjected to statistical analysis.

### 2.11 Histological tissue section preparation

Kidneys and brainstems were excised post-euthanasia and immediately placed in 4%-paraformaldehyde for 24-h at 4°C (kidneys cut transversely in half). Tissue was then stored in a 70% ethanol solution at 4°C until embedding. Before paraffin embedding of the brain, a transverse cut was made at the level of the rostral medulla/caudal pons, suggested as the optimal location for obtaining sections containing the RTN (Lavezzi et al., 2019). Tissue was embedded in paraffin blocks using standard embedding procedures. Briefly, tissue was dehydrated through incubations in 80% and 95% ethanol, 45 min each, and followed by 3 changes of 100% ethanol, 1 h each. Tissue was cleared through 2 changes of xylene, 1 h each, and placed in molten paraffin overnight (H-PF, General Data, Cincinnati, OH). Tissue was then embedded, cut side down, in paraffin blocks. Tissue was sectioned at a thickness of 5 μm, mounted on frosted slides, and dried at 37°C overnight.

### 2.12 Immunohistochemistry and histological staining

Standard immunoperoxidase procedures were used for chromogenic immunostaining. Briefly, sections were deparaffinized in xylene, rehydrated in decreasing ethanol concentrations, and rinsed in cool tap water. Sections were incubated in a Tris/EDTA solution (in mM: 10 Tris-base, 1 EDTA; pH 9–9.1) at 95°C for 40 min and let cooled at room temperature for 20 min. Endogenous peroxidase activity was blocked using Peroxidase Suppressor (35000, Thermo Fisher Scientific) for 10 min. Sections were rinsed 2x for 3 min in TBS with 0.05% Tween 20 (0.05% TBS-T). The sections were blocked with Rodent Block M (for NBCe1-B and Phox2B staining; Biocare Medical, Pacheco, CA) or 10% normal goat serum (for PEPCK and GS staining; Thermo Fisher Scientific) for 30 min. For PEPCK and GS staining, sections were additionally permeabilized in 0.5% Triton X-100 prepared in TBS for 15 min. Sections were incubated overnight in a humidified chamber at 4°C with primary antibody dilutions prepared in a TBS solution with 5% BSA (5% TBS-BSA). The next day, sections were washed 3x 5-min with 0.05% TBS-T. Sections were incubated with HRP-conjugated secondary antibody diluted in 5% TBS-BSA for 1 h at room temperature, and again washed 3x 5-min with 0.05% TBS-T. Sections were then exposed to diaminobenzidine (DAB) for 10 min. To counterstain nuclei, sections were rinsed with DI water and stained with hematoxylin (Abcam; hematoxylin ab220365) for 1 min and rinsed with DI water. All sections were dehydrated with graded ethanol solutions and xylene, and mounted for light microscopy. Comparisons of labeling were made only between sections from the same immunohistochemistry experiment, treated with identical reagents. In some replicates, sections were included that were only treated with primary or secondary antibodies as internal controls. Images were taken with a Leica DM 6B upright microscope with identical capture settings between slides from the same experiment. For analysis of NBCe1-B/C expression intensity in brainstem IHC images in Figure 2, images were converted to 8-bit greyscale images and inverted, and the average pixel intensity was measured within a region of interest (ROI) containing the tissue section using FIJI. Similar measurements were made for a background ROI, which was subtracted from the tissue section ROI measurement. For quantification of PEPCK and GS expression in Figures 8, 9, high magnified images were taken across the cortical and OSOM regions (5-images per region). Individual PTs were manually traced (identified by the presence of a prominent apical brush border; in contrast, distal tubules have sparse microvilli, which makes the lumen appear to be wider), and images were converted to 8-bit greyscale images and inverted. A 30–255-bit threshold was applied to all images in order to limit inclusion of background pixels, and the mean pixel intensity of selected tubules was measured. The average pixel intensity across all 5 images within the cortical or OSOM regions was normalized to the average in the WT cortex.

### 2.13 Statistical analysis

Results are presented as mean ± SEM, with n referring to the number of animals studied. In all analyses the threshold of *p* < 0.05 was used to determine statistical significance. Normality of data was tested in GraphPad (v9.0) using the D'Agostino-Pearson omnibus test and statistical comparisons between two groups (i.e., WT and KO_b/c_ mice) with normal distributions were performed using Student’s two-tailed unpaired *t*-test. Non-normal distributions were only observed in the control NH_4_^+^ excretion data set (Figure 7A, left) and thus for these comparisons only, the Mann-Whitney test (2-tailed, unpaired) was used. *p*-values were Bonferroni corrected for multiple comparisons when appropriate. For plethysmography and blood gas experiments (Figures 5, 6), in which repeated measures in individual mice were possible, repeated measures ANOVA (RM-ANOVA) was used to assess the response of WT and KO_b/c_ groups to MAc, while comparisons between WT and KO_b/c_ mice at each timepoint was assessed using Student’s unpaired two-tailed *t*-test. Since in these experiments the percent change from baseline was also calculated, animals with outliers (defined as+/-2 SD from the mean) in the baseline (timepoint “0”) data set only, were excluded from analysis; which equated to 1 animal from the plethysmography data set, 1 animal from the pCO_2_ data set, and 2 animals from the plasma pH data set being excluded. Conversely, assessment of plasma electrolytes required sacrificing mice after 1-, 2-, or 3-days of control or MAc-challenged conditions; thus each day represents a different cohort of mice. However, in these same experiments, metabolic and urinary acid-excretion parameters were recorded daily, leading to sequentially larger sample sizes for day 1, then day 2 or day 3. Thus, comparison between WT and KO_b/c_ metabolic and urinary acid-parameters over the 3-day experimental time course were assessed by 2-way ANOVA, using genotype and time as independent variables, with the main effect of genotype specifically reported (“G”, Table 2 and Figure 7). For urinary parameters in Figure 7, the genotype x time (“G*T”) interaction effect is also reported and comparison of WT and KO_b/c_ responses were assessed at each timepoint using Student’s unpaired two-tailed *t*-test. Both male and female mice were used non-discriminately throughout the study based on availability at the time of each experiment. Inclusion of sex as an independent factor in ANOVA was used to assess for a significant effect of sex; if a significant sex interaction was found this is reported specifically. All analyses were performed in Prism GraphPad version 9 or IBM SPSS. Figures were prepared using Microsoft PowerPoint, Microsoft Excel, BioRender.com, and Prism GraphPad version 9.

## 3 Results

### 3.1 Validation of an NBCe1-B/C specific antibody

**FIGURE 1:**
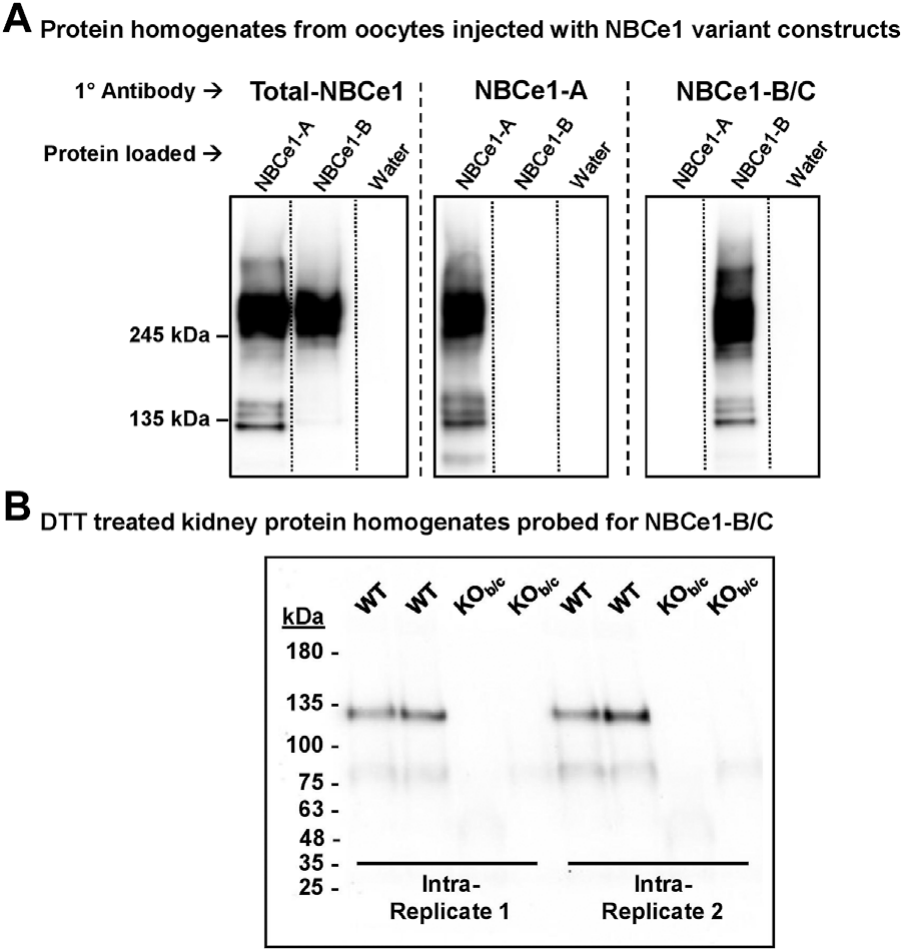
Validation of an NBCe1-B/C specific antibody (Ab). **(A)** Specificity of the NBCe1-B/C Ab. Protein homogenates from human NBCe1-A-EGFP cRNA, human NBCe1-B-EGFP cRNA, or water injected oocytes were loaded in triplicate on a single gel. After protein transfer, the blot was cut into three pieces and probed with either an anti-Total-NBCe1 Ab (left), an anti-NBCe1-A-specific Ab (middle), or an anti-NBCe1-B/C-specific Ab (right). In the anti-Total-NBCe1-Ab-probed blot, bands were observed in the NBCe1-A-EGFP and NBCe1-B-EGFP protein loaded lanes, corresponding with the predicted molecular weights of the monomers and dimerized NBCe1-A-EGFP and NBCe1-B-EGFP variants. In the blots probed with the anti-NBCe1-A or anti-NBCe1-B/C specific Abs, these bands were only observed in the lane corresponding with the applied Ab. **(B)** Use of NBCe1-B/C Ab in WT and KO_b/c_ purified protein homogenates. Homogenates were treated with 0.1M dithiothreitol (DTT) to reduced endogenous mouse immunoglobulin that would otherwise interfere with measurement of NBCe1-B abundance. The presence of the band consistent with NBCe1-B monomer abundance in WT, but not KO_b/c_, kidney protein homogenates, confirms the validity of the NBCe1-B/C Ab in detecting NBCe1-B in the kidney.

Protein homogenates prepared from NBCe1-A-EGFP cRNA-, NBCe1-B-EGFP cRNA-, or water- injected oocytes were loaded on a single gel, in triplicate. After protein transfer, the PVDF membrane was cut into thirds, and antibodies (Abs) specific to Total-NBCe1 (affinity for all NBCe1 variants), NBCe1-A, or NBCe1-B/C were used to individually probe the three membrane sections (Figure 1A). The anti-Total-NBCe1-Ab-probed blots reported bands that are consistent with the molecular weights of NBCe1-EGFP monomers and dimers (Parker et al., 2012) in both the NBCe1-A-EGFP and NBCe1-B-EGFP-loaded lanes (Figure 1A, left). The anti-NBCe1-A and anti-NBCe1-B/C-Ab-probed blots only reported bands in the lanes loaded with NBCe1-A-EGFP or NBCe1-B-EGFP protein, respectively (Figure 1A, center and right).

To assess the validity of the NBCe1-B/C specific antibody in mouse kidney preparations (where NBCe1-A and NBCe1-B are expressed, but NBCe1-C is not), we probed for NBCe1-B in WT and KO_b/c_ kidney protein homogenates treated with 0.1M dithiothreitol (DTT; see ‘Methods–Western blotting’ for rationale; Figure 1B). We detect immunoreactivity consistent with monomeric NBCe1-B in homogenates from WT but not from KO_b/c_ mice. Thus the antibody is appropriate for specific detection of NBCe1-B in the kidney.

### 3.2 Expression of NBCe1-B/C in the brainstem of WT and KO_b/c_ mice

**FIGURE 2:**
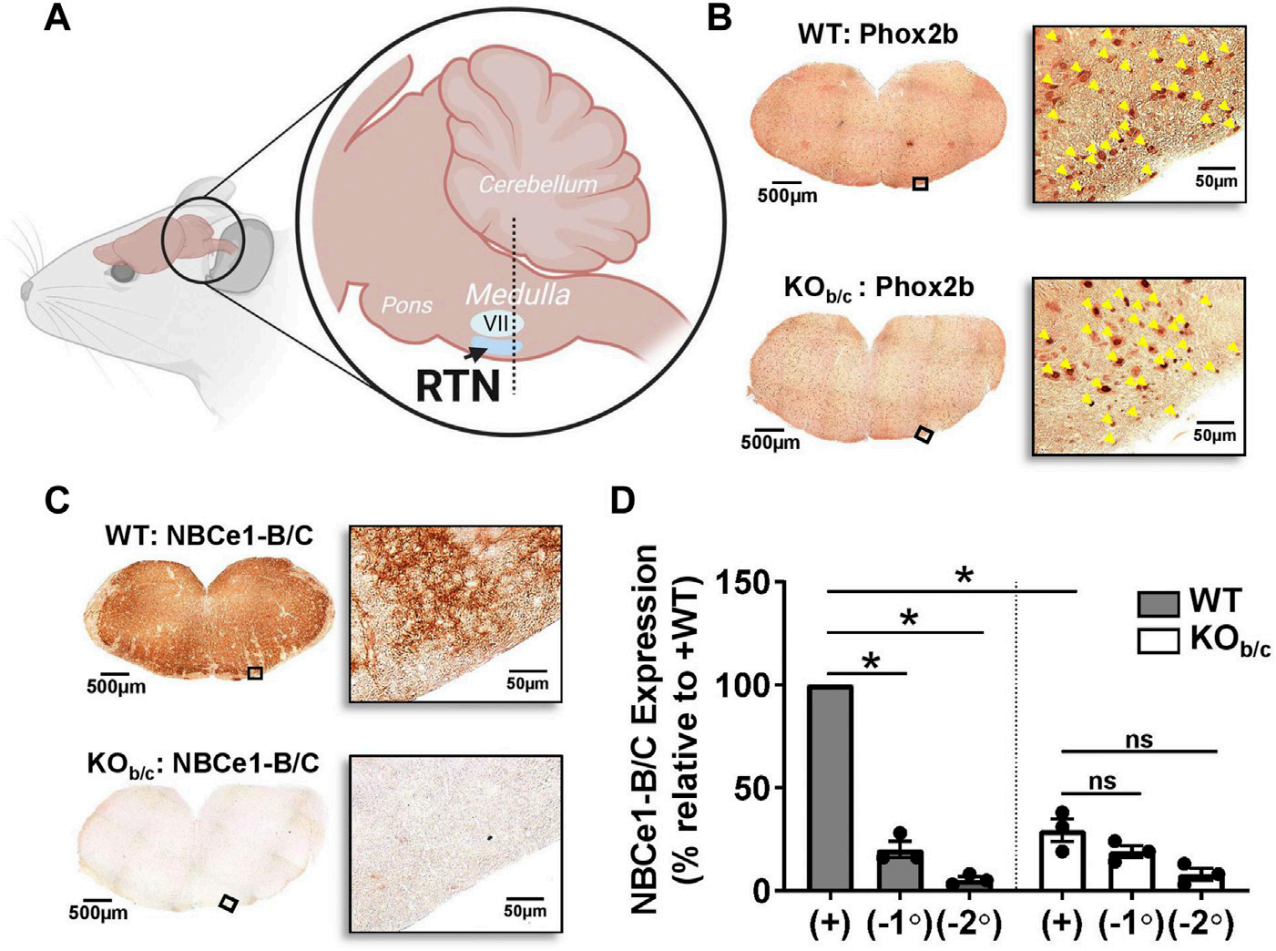
Absence of NBCe1-B/C expression in the brainstem medulla of KO_b/c_ mice. **(A)** Cartoon illustration of murine brainstem, demonstrating the location (dotted line) from which medullary brainstem sections were obtained, which is suggested to be the ideal sampling location for sections containing the retrotrapezoid nucleus (RTN; VII, facial nucleus). **(B)** Tiled and higher magnified regions of interest on the ventral portion of WT and KO_b/c_ medullary brainstem sections that were stained with Phox2B, a transcription factor expressed in RTN neurons. Yellow arrowheads in magnified images signify Phox2B expressing nuclei, which are present in both WT and KO_b/c_ medullary brainstem sections, indicating that Phox2B expressing neurons are intact in KO_b/c_ mice. **(C)** Tiled and higher magnified regions of interest of WT and KO_b/c_ medullary brainstem sections stained with NBCe1-B/C, confirming the absence of NBCe1-B/C in KO_b/c_ mice. **(D)** Quantification of NBCe1-B/C immunolabeling in 3 pairs of WT and KO_b/c_ sections that included negative control sections in which application of the primary (−1°) or secondary (−2°) antibody was excluded. The intensity of immunolabeling in KO_b/c_ sections is significantly less than in WT but is not significantly different from the intensity of the negative control sections. *p < 0.05 by Student’s 2-tailed unpaired *t*-test with Bonferroni correction for multiple comparisons; ns, non-significant.

Before testing the hypothesis that NBCe1-B/C has a role in the respiratory response to acidosis we first confirmed the loss of NBCe1-B/C in the brainstem medulla of KO_b/c_ mice. Sections were cut from the rostral medulla of the brainstem, starting from the dotted line illustrated in Figure 2A and moving rostrally, which is suggested to be the optimal sampling location for sampling the RTN (Lavezzi et al., 2019). The images in Figure 2B demonstrate Phox2B expression (yellow arrowheads), a transcription factor expressed in RTN neurons (Stornetta et al., 2006; Dubreuil et al., 2008; Ruffault et al., 2015), in both WT (top) and KO_b/c_ (bottom) medullary brainstem sections. Thus, Phox2B- expressing neurons appear intact in the brainstem medulla of KO_b/c_ mice. Figure 2C shows representative images of medullary brainstem sections from WT (top) and KO_b/c_ (bottom) mice probed with the NBCe1-B/C antibody, showing absence of NBCe1-B/C immunoreactivity in brainstem medulla of KO_b/c_ mice. Figure 2D summarizes the results of three experimental replicates in which the intensity of NBCe1-B/C immunolabel was quantified in identically treated/imaged WT and KO_b/c_ sections, with the percent of NBCe1-B/C expression in KO_b/c_ sections calculated relative to WT. Similar calculations were made for negative control sections in which either the primary antibody or the secondary antibody was excluded (−1° and −2°, respectively; images not shown). The chromogenic signal intensity in KO_b/c_ sections treated with the NBCe1-B/C antibody was significantly less than in WT sections (*p* = 0.015, Bonferroni corrected), and was not significantly different from either of the negative controls, confirming the absence of majority NBCe1-B/C expression in the brainstem of KO_b/c_ mice.

### 3.3 Expression of kidney NBCe1-A and NBCe1-B under control and MAc-challenged conditions

The original characterization of NBCe1-B expression in the kidney was in the context of the congenitally acidemic NBCe1-A KO mouse, and since NBCe1-B expression is controlled by an acid-sensitive promoter (Snead et al., 2011), the NBCe1-B expression described in NBCe1-A KO mice may not be representative of expression in WT mice. Therefore, we aimed to determine the expression pattern and abundance response of NBCe1-B in WT mice at baseline and during MAc. We also investigated the abundance response of NBCe1-A during MAc in both WT and KO_b/c_ mice.

**FIGURE 3:**
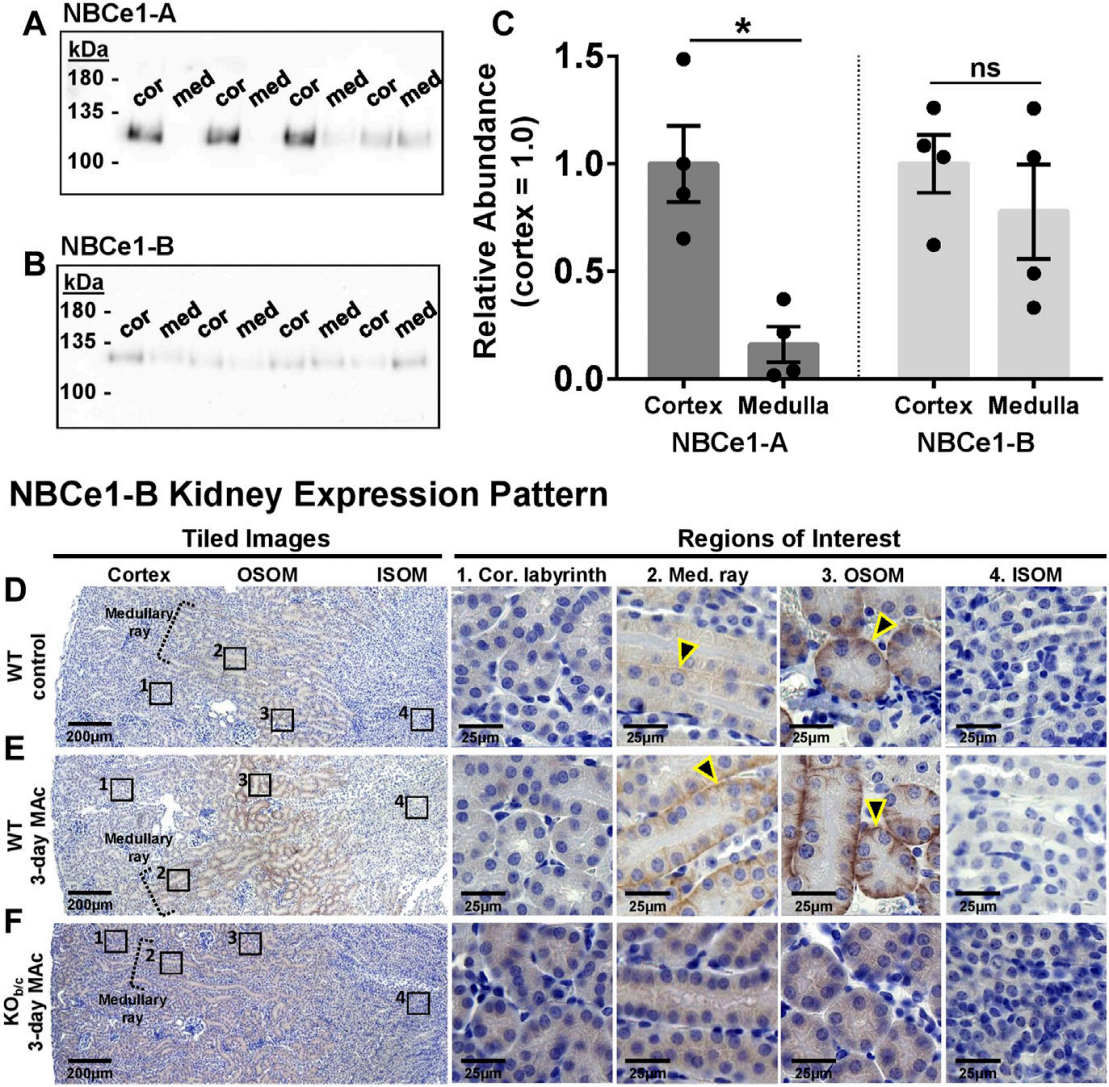
NBCe1-A and NBCe1-B kidney expression. **(A,B)** Immunoblot analysis of protein preparations from micro-dissected cortex (cor) and medulla (med) from WT mice. Two gels were identically loaded (2 µg/lane) and probed with the anti-NBCe1-A **(A)** or anti-NBCe1-B/C **(B)** antibody. **(C)** Quantification of Western blot results, with medullary abundance normalized to average cortical abundance. There was significantly more NBCe1-A abundance in the cortex than medulla, whereas there was no difference in NBCe1-B abundance between the cortex and medulla (n = 4). **(D–F)** Immunohistochemistry examining NBCe1-B immunolabeling in the cortical labyrinth, cortical medullary ray, outer stripe of outer medulla (OSOM), and inner stripe of outer medulla (ISOM). Left-hand images are tiled images, and right-hand images are higher magnified regions of interest. **(D)** Under control conditions, NBCe1-B was detectable in the basolateral membrane of PTs located in the cortical medullary ray and OSOM (arrowheads), but was not detectable in PTs of the cortical labyrinth or in the ISOM. **(E)** After 3 days of MAc, NBCe1-B immunolabel intensity qualitatively increased in PTs located in the cortical medullary ray and OSOM (arrowheads). **(F)** No basolateral NBCe1-B immunoreactivity was observed in KO_b/c_ mice after 3 days of MAc. Images are representative of 3 replicate experiments each. *p < 0.05 by Student’s 2-tailed unpaired *t*-test; ns, non-significant.

Using the NBCe1-A and NBCe1-B/C specific antibodies described in Figure 1, we first assessed the abundance of NBCe1-A and NBCe1-B in WT cortical (cor) and medullary (med) protein preparations by Western blot (Figures 3A, B). Although the NBCe1-B/C specific antibody could also recognize NBCe1-C, only NBCe1-B is expressed in the kidney (Fang et al., 2018). Since these preparations contained less protein compared to whole kidney lysates, in this experiment two µg of protein was loaded per lane. The Memcode total protein stain was used to normalize protein loading/transfer among lanes (data not shown). We found, as expected, that NBCe1-A abundance is significantly greater in the cortex than medulla (*p* = 0.002; Figure 3C, left). On the other hand, NBCe1-B abundance was not significantly different between the cortex and medulla (Figure 3C, right). To determine the specific location of NBCe1-B expression in WT kidneys we used immunohistochemistry (Figures 3D–F, n = 3 replicates for each). In kidney sections from control WT mice, we observed NBCe1-B immunoreactivity on the basolateral membrane of PTs located in cortical medullary rays, as well as in PTs of the OSOM (Figure 3D; yellow outlined arrowheads indicate representative tubules that have positive basolateral immunoreactivity). After 3-days of MAc, we qualitatively observed a greater intensity of basolateral NBCe1-B staining in the PTs of the cortical medullary ray and OSOM (Figure 3E). As a control, basolateral NBCe1-B immunoreactivity was absent in KO_b/c_ mice even after 3 days of MAc (Figure 3F, n = 3 replicates). Together, these data indicate that NBCe1-B in WT mice is expressed primarily in the PTs of the cortical medullary ray and OSOM, with no obvious expression detectable in PTs of the cortical labyrinth, even after 3-days of MAc-challenged conditions.

**FIGURE 4:**
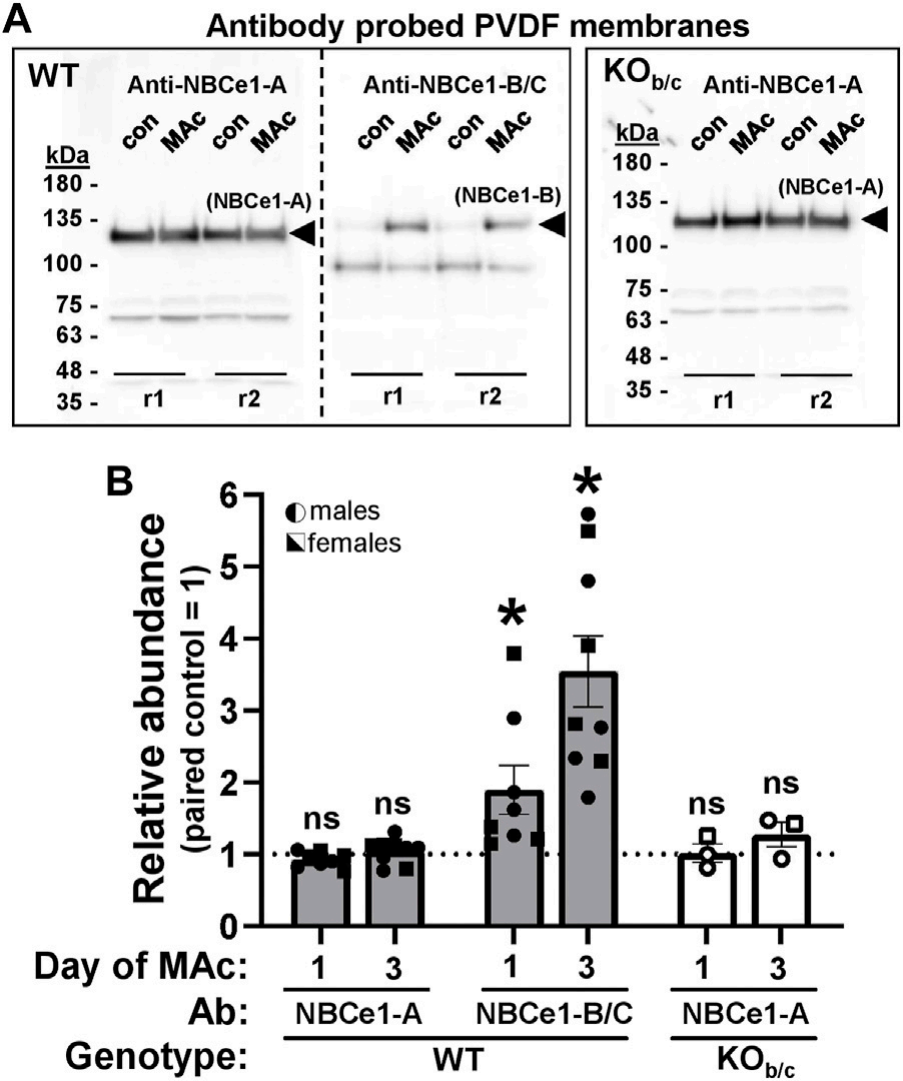
Abundance response of renal NBCe1-A and NBCe1-B during MAc. **(A)** Chemiluminescent images of western blots, loaded with 10 µg/lane of protein prepared from paired, sex-matched, littermates kept under control (con) or 3-days of metabolic acidosis (MAc) conditions. Two intra-assay replicates for each mouse pair (r1 and r2) were included in each experiment. For WT, each set of replicates was loaded twice for probing with either an NBCe1-A or NBCe1-B specific antibody (left and right sides of blot). For KO_b/c_, replicates were only loaded once for probing with the NBCe1-A specific antibody. Bands representing NBCe1-A and NBCe1-B abundance are observed at ∼125 kDa. The lower molecular weight (∼100 kDa) bands in the anti-NBCe1-B/C treated blot are endogenous mouse immunoglobulin subunits resulting from DTT reduction of intact immunoglobulin (Ahmad-Zadeh et al., 1971), which would otherwise interfere with measurement of NBCe1-B abundance. We are unsure of the identity of the ∼70 kDa band in the anti-NBCe1-A treated blots. **(B)** Quantification of NBCe1-A and NBCe1-B abundance in WT mice, and NBCe1-A abundance in KO_b/c_ mice, after 1- or 3-days of MAc. ‘Relative Abundance’ during MAc is relative to that of a paired, sex-matched, littermate subjected to control conditions, such that each point in Figure 4B represents a single MAc/control pair (i.e., control = 1, represented by the dotted line). *p < 0.05 by Student’s 2-tailed unpaired *t*-test; ns, non-significant; WT 1-day: n = 4M/4F, 3-day: n = 5M/4F; KO_b/c_: n = 2M/1F for both 1- and 3-day.

Next, we assessed the change in protein abundance of both NBCe1-A and NBCe1-B variants in the kidneys of WT mice, as well as the change in NBCe1-A kidney abundance in KO_b/c_ mice, during MAc. In these experiments, sex-matched littermates were paired, with one subjected to control conditions (con) and the other to MAc-challenged conditions for 1 or 3 days. For Western blotting, 10 µg of kidney lysate protein was loaded per lane. Prior to antibody application each blot was treated with the reversible Memcode total-protein stain in order to provide an index for normalizing protein loading/transfer among lanes (data not shown). The WT membrane was then cut, and probed with either the NBCe1-A or NBCe1-B specific antibody, while the KO_b/c_ membrane was probed with just the NBCe1-A specific antibody. Figure 4A shows a representative Western blot from a 3-day experiment demonstrating NBCe1 monomer immunoreactivity. NBCe1 abundance (from Western blot) was normalized to total-protein abundance (from Memcode stain) and the ratio of this normalized abundance was calculated between paired MAc-challenged and control littermates; thus, each point in Figure 4B represents a single MAc/control pair (i.e., control = 1, represented by the dotted line in Figure 4B). In WT mice, after 1 or 3 days of MAc, NBCe1-A abundance was not significantly different from control (Figure 4B). On the other hand, in the same WT mice, NBCe1-B abundance was significantly increased after both 1- and 3-days of MAc (1-day ratio, *p* = 0.033; 3-day ratio, *p* < 0.001; Figure 4B). Finally, similar to WT mice, there was no significant change in NBCe1-A abundance in KO_b/c_ mice after 1- or 3-days of MAc (Figure 4B). Overall, this data indicates that NBCe1-B is expressed in the PTs of the medullary and OSOM in WT mice and that the abundance of NBCe1-B, but not NBCe1-A, increases during MAc.

### 3.4 Respiratory response of KO_b/c_ mice during MAc

**FIGURE 5:**
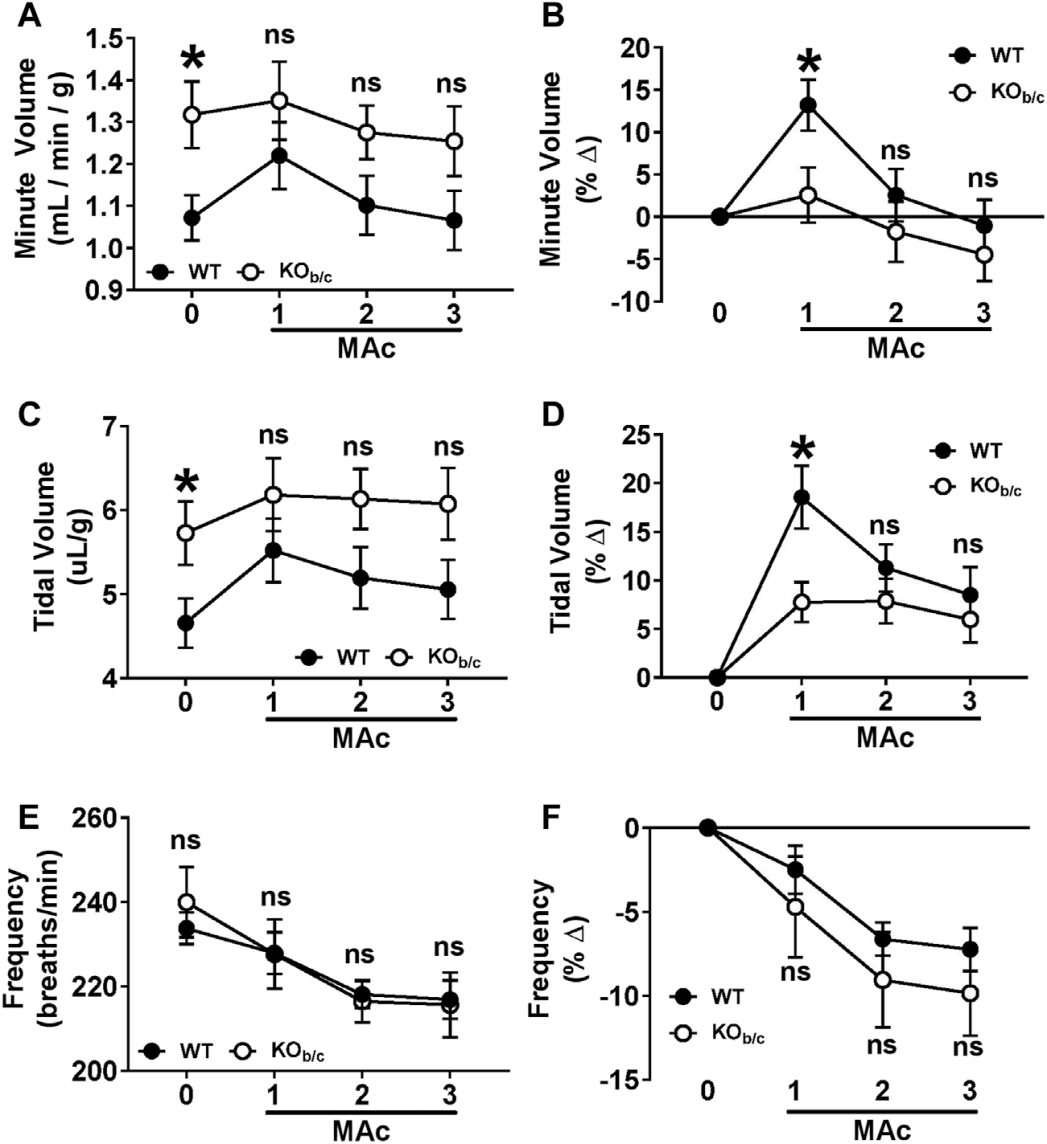
Ventilation response of KO_b/c_ mice during 3-day MAc-challenge. Minute volume **(A,B)**, tidal volume **(C,D)**, and frequency **(E,F)** were measured for 2-days under non-challenged conditions and averaged for use as baseline data (timepoint “0”), followed by 3-days of MAc-challenged conditions (timepoints “1–3”). Panels A, C, and E show the averaged parameter values, and panels B, D, and F show the average % change from baseline, for each genotype after each day of the 3-day MAc-challenge. **(A)** Minute volume. At baseline, KO_b/c_ minute volume was significantly higher than WT (timepoint 0), but there were no significant differences during MAc (timepoints 1–3). **(B)** When normalized to baseline, WT mice exhibited a significant % change in minute volume in response to MAc (*p* < 0.001, RM-ANOVA) that was not observed in KO_b/c_ mice (*p* = 0.227, RM-ANOVA). Furthermore, the % change in minute volume after 1 day of Mac was significantly greater in WT mice than KO_b/c_ mice (timepoint 1). **(C)** Tidal Volume. At baseline, KO_b/c_ tidal volume was significantly higher than WT (timepoint 0), but there were no differences during MAc (timepoints 1–3). **(D)** When normalized to baseline, both WT and KO_b/c_ mice exhibited a significant % change in tidal volume in response to MAc (WT: *p* < 0.001, KO_b/c_: *p* = 0.007; RM-ANOVA), but the % change in tidal volume after 1 day of MAc was significantly greater in WT mice than KO_b/c_ mice (timepoint 1). **(E)** Frequency. There were no significant differences between WT and KO_b/c_ frequencies at baseline or during the MAc-challenge. **(F)** When normalized to baseline, both WT and KO_b/c_ mice exhibited a significant % change in frequency in response to MAc (WT: *p* < 0.001, KO_b/c_: *p* = 0.007; RM-ANOVA). There were no significant differences in % change in frequency between WT and KO_b/c_ mice on any day of the MAc-challenge. *p < 0.05 between WT and KO_b/c_ mice at each day noted, assessed by Student’s 2-tailed unpaired *t*-test; ns, non-significant; WT: n = 5M/6F; KO_b/c_: n = 7M/4F.

Next, we assessed the ventilation of WT and KO_b/c_ mice prior to, and during each day of, a 3-day MAc-challenge using unrestrained whole-body plethysmography. Ventilation parameters (minute volume, tidal volume, and frequency) of WT and KO_b/c_ mice were assessed for 2 days under unchallenged conditions and averaged to establish a baseline (Figures 5A–F, timepoint “0”), followed by repeated measures after each 24-h period of a 3-day MAc-challenge (Figures 5A–F, timepoints “1–3”). Minute volume and tidal volume were corrected for body-weight in individual mice. Figures 5A,C,E show the average values for WT and KO_b/c_ groups at each timepoint. At baseline, KO_b/c_ mice had a significantly higher minute volume than WT (*p* = 0.019; Figure 5A, timepoint 0) due to a significantly greater baseline tidal volume (*p* = 0.038; Figure 5C, timepoint 0), while there was no significant difference in baseline frequency (Figure 5E, timepoint 0). There were no significant differences in the values of these parameters for the duration of the 3-day MAc-challenge (Figures 3A,C,E, timepoints 1–3).

Since there were significant baseline differences in ventilation parameters between WT and KO_b/c_ mice, we assessed the respiratory response of WT and KO_b/c_ mice to MAc by calculating the percent change from baseline of each ventilatory parameter during the 3-day MAc-challenge (Figures 3B,D,F). MAc had a significant effect on WT minute volume (*p* < 0.001 by RM-ANOVA; Figure 3B) but had no significant effect on KO_b/c_ minute volume (*p* = 0.227 by RM-ANOVA; Figure 3B). Furthermore, after the first day of MAc, pairwise comparisons demonstrate a significantly greater percent increase in minute volume in WT mice compared to KO_b/c_ mice (*p* = 0.026; Figure 5B, timepoint 1). MAc had a significant effect on tidal volume in both WT and KO_b/c_ mice (WT: *p* < 0.001, KO_b/c_: *p* = 0.007 by RM-ANOVA; Figure 5D), however WT mice had a significantly greater percent increase in tidal volume compared to KO_b/c_ mice after the first day of MAc (*p* = 0.010; Figure 5D, timepoint 1). Lastly, MAc had a significant effect on respiratory frequency in both WT and KO_b/c_ mice (WT: *p* < 0.001, KO_b/c_: *p* = 0.007 by RM-ANOVA; Figure 5F), but there were no significant differences in the percent change in frequency between WT and KO_b/c_ mice at any timepoint (Figure 5F). Overall, the absence of an increase in minute volume and diminished increase in tidal volume after 1 day of MAc in KO_b/c_ mice is consistent with the hypothesis that the respiratory response to MAc is impaired in KO_b/c_ mice.

### 3.5 Acid-base status of KO_b/c_ mice during MAc

**FIGURE 6:**
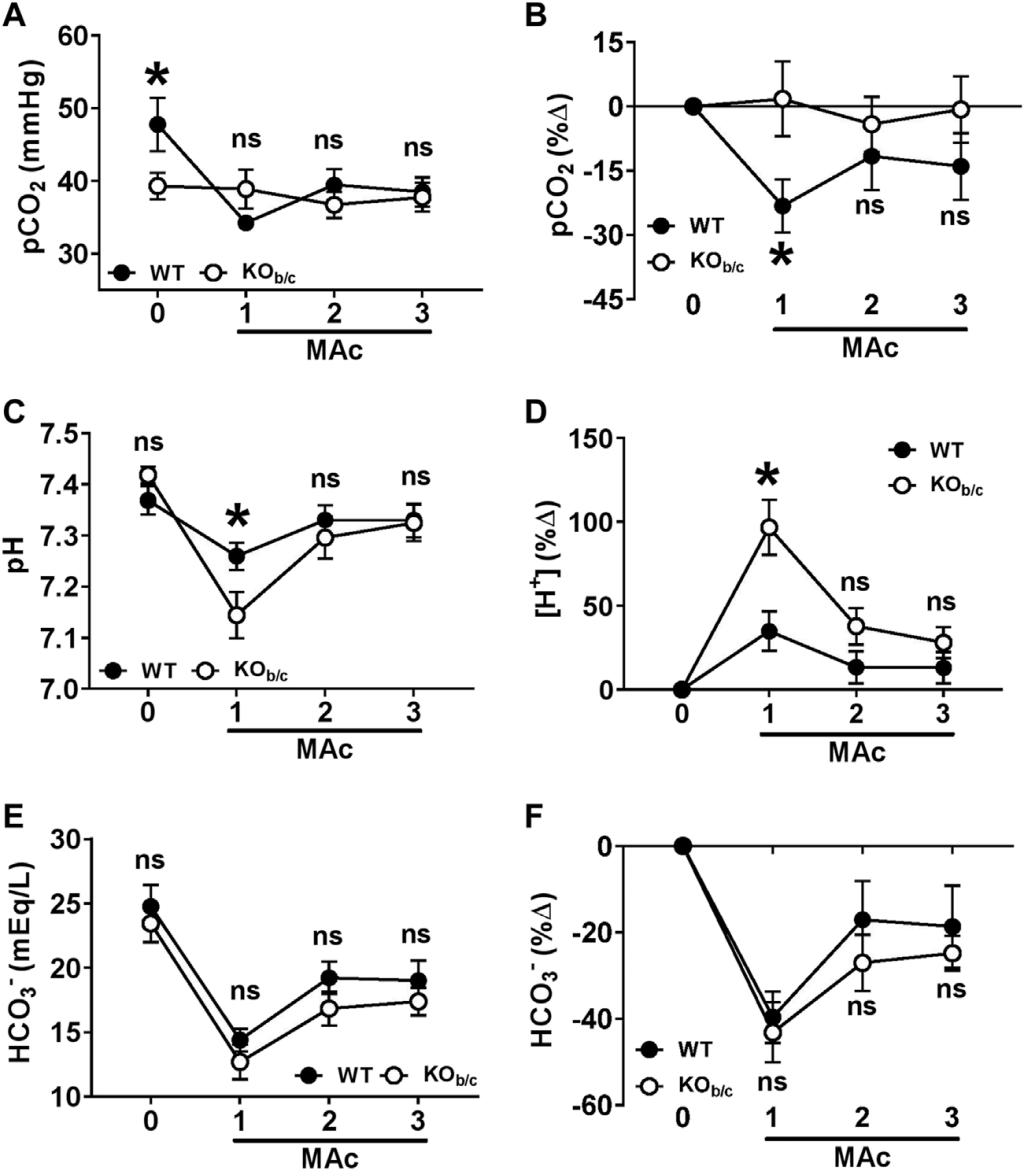
**A**cid-base status of KO_b/c_ mice during 3-day MAc-challenge. pCO_2_ **(A,B)**, plasma pH **(C,D)**, and [HCO_3_^−^] **(E,F)**, were measured in unanesthetized animals the day before (timepoint “0”) and each day of a 3-day MAc-challenge (timepoints “1–3”). Panels A, C, and E show the averaged parameter values, and panels B, D, and F show the average % change from baseline, for each genotype after each day of the 3-day MAc-challenge. **(A)** pCO_2_. At baseline, KO_b/c_ mice had a significantly lower pCO_2_ than WT mice (timepoint 0) but there were no significant differences during MAc (timepoints 1–3). **(B)** When normalized to baseline, WT mice exhibited a significant % change in pCO_2_ in response to MAc (*p* = 0.011, RM-ANOVA) that was not observed in KO_b/c_ mice (*p* = 0.889, RM-ANOVA). Furthermore, the % decrease in pCO_2_ in WT mice after 1 day of MAc was significantly different from the % change in KO_b/c_ mice (timepoint 1). **(C)** Plasma pH. There were no significant differences between the plasma pH of WT and KO_b/c_ at baseline (timepoint 0), but after 1 day of MAc the pH of KO_b/c_ mice was significantly less than WT mice (timepoint 1). **(D)** For assessing % change from baseline, pH was converted to [H^+^]. When normalized to baseline, both WT and KO_b/c_ mice exhibited a significant % change in [H^+^] in response to MAc (WT: *p* = 0.039, KO_b/c_: *p* < 0.001; RM-ANOVA), however the % change in [H^+^] after 1 day of MAc was significantly greater in KO_b/c_ mice than WT mice (timepoint 1). **(E)** [HCO_3_^−^]. There were no significant differences between WT and KO_b/c_ [HCO_3_^−^] at baseline or during the MAc challenge. **(F)** When normalized to baseline, both WT and KO_b/c_ mice exhibited a significant % change in [HCO_3_^−^] in response to MAc (WT: *p* < 0.001, KO_b/c_: *p* < 0.001; RM-ANOVA), but there were no significant differences in the % change in [HCO_3_^−^] on any day of the MAc-challenge. *p < 0.05 between WT and KO_b/c_ mice at each day noted, assessed by Student’s 2-tailed unpaired *t*-test; ns, non-significant; WT: n = 7M/6F; KO_b/c_: n = 6M/8F.

To determine the effect of NBCe1-B/C loss on the defense of acid-base status during MAc, we assessed the pCO_2_, pH, and [HCO_3_^−^] of WT and KO_b/c_ mice at baseline (Figures 6A–F, timepoint “0”) followed by repeated measures after each 24-h period of a 3-day MAc-challenge (Figures 6A–F, timepoints “1–3”). Figures 6A,C,E show the average values for WT and KO_b/c_ groups at each timepoint. At baseline, KO_b/c_ mice had a significantly lower pCO_2_ than WT mice (*p* = 0.046; Figure 6A, timepoint 0), but had no significant difference from WT in baseline pH or baseline [HCO_3_^−^] (Figures 6C, E; timepoint 0). During the 3-day MAc-challenge, there were no significant differences between WT and KO_b/c_ pCO_2_ or [HCO_3_^−^] at any timepoint (Figures 6A–E, timepoints 1–3), however KO_b/c_ pH was significantly lower than WT after the first day of MAc (*p* = 0.038; Figure 6C, timepoint 1).

Since there was a baseline difference in pCO_2_ between WT and KO_b/c_ mice, we assessed the recovery of acid-base status in WT and KO_b/c_ mice during MAc by calculating the percent change from baseline in each parameter during the 3-day MAc-challenge (Figures 6B,D,F). For plasma pH, we transformed all values to hydrogen ion concentration ([H^+^]) in order to directly calculate the percent change in acidity from baseline (Figure 6D). MAc had a significant effect on pCO_2_ in WT mice (*p* = 0.011 by RM-ANOVA; Figure 6B) but had no effect on pCO_2_ in KO_b/c_ mice (*p* = 0.889 by RM-ANOVA; Figure 6B). Furthermore, after 1-day of MAc, pairwise comparisons demonstrate a significantly greater percent decrease in pCO_2_ in WT mice compared to KO_b/c_ mice (*p* = 0.029; Figure 6B, timepoint 1). MAc had a significant effect on [H^+^] in both WT and KO_b/c_ mice (WT: *p* = 0.039, KO_b/c_: *p* < 0.001 by RM-ANOVA; Figure 6D), however the increase in [H^+^] after the first day of MAc was significantly greater in KO_b/c_ mice than in WT mice (*p* = 0.005; Figure 6D, timepoint 1). Lastly, MAc had a significant effect on [HCO_3_^−^] in both WT and KO_b/c_ mice (WT: *p* < 0.001, KO_b/c_: *p* < 0.001 by RM-ANOVA; Figure 6F), and there were no significant differences in the percent change from baseline [HCO_3_^−^] between WT and KO_b/c_ mice at any timepoint (Figure 6F). In summary, the lack of a decrease in pCO_2_ in KO_b/c_ mice appears to underlie a greater fall in plasma pH after 1-day of MAc but does not inhibit the recovery of pH after 2- and 3-days of MAc.

### 3.6 Renal response of KO_b/c_ mice during MAc

Since we observed a recovery of plasma pH in KO_b/c_ mice to a level similar to that in WT mice, despite KO_b/c_ mice having an impaired respiratory response (Figure 5) and more severe acidemia (Figure 6) after 1 day of MAc, we hypothesized that renal acid-excretion is enhanced in KO_b/c_ mice during the MAc-challenge. To test this hypothesis, we subjected WT and KO_b/c_ mice to control or MAc-challenged conditions for 1, 2, or 3 days while housed in metabolic cages and assessed daily 24-h urine collections for NH_4_^+^ excretion, titratable acid (TA) excretion, and pH. Additionally, mice were sacrificed after each timepoint via cardiac puncture for assessment of electrolytes. As expected, mice subjected to MAc-challenged conditions exhibited significantly greater NH_4_^+^ and TA excretion, and significantly lower urinary pH than animals of the same genotype under control conditions (*p* < 0.001 for all three parameters in both genotypes by ANOVA). Acid-excretion parameters were compared between WT and KO_b/c_ groups using 2-way ANOVA with genotype and time as independent variables. A significant main effect of genotype (“G”) indicates a significant difference between overall group means (i.e., average daily NH_4_^+^ excretion between WT and KO_b/c_ mice) and a significant genotype x time (“G*T”) interaction effect indicates that the difference between genotypes depends on time. Thus, pairwise comparisons between WT and KO_b/c_ groups were assessed at each timepoint; for transparency, the significance of pairwise comparisons are reported for all parameters regardless of G*T significance.

**FIGURE 7:**
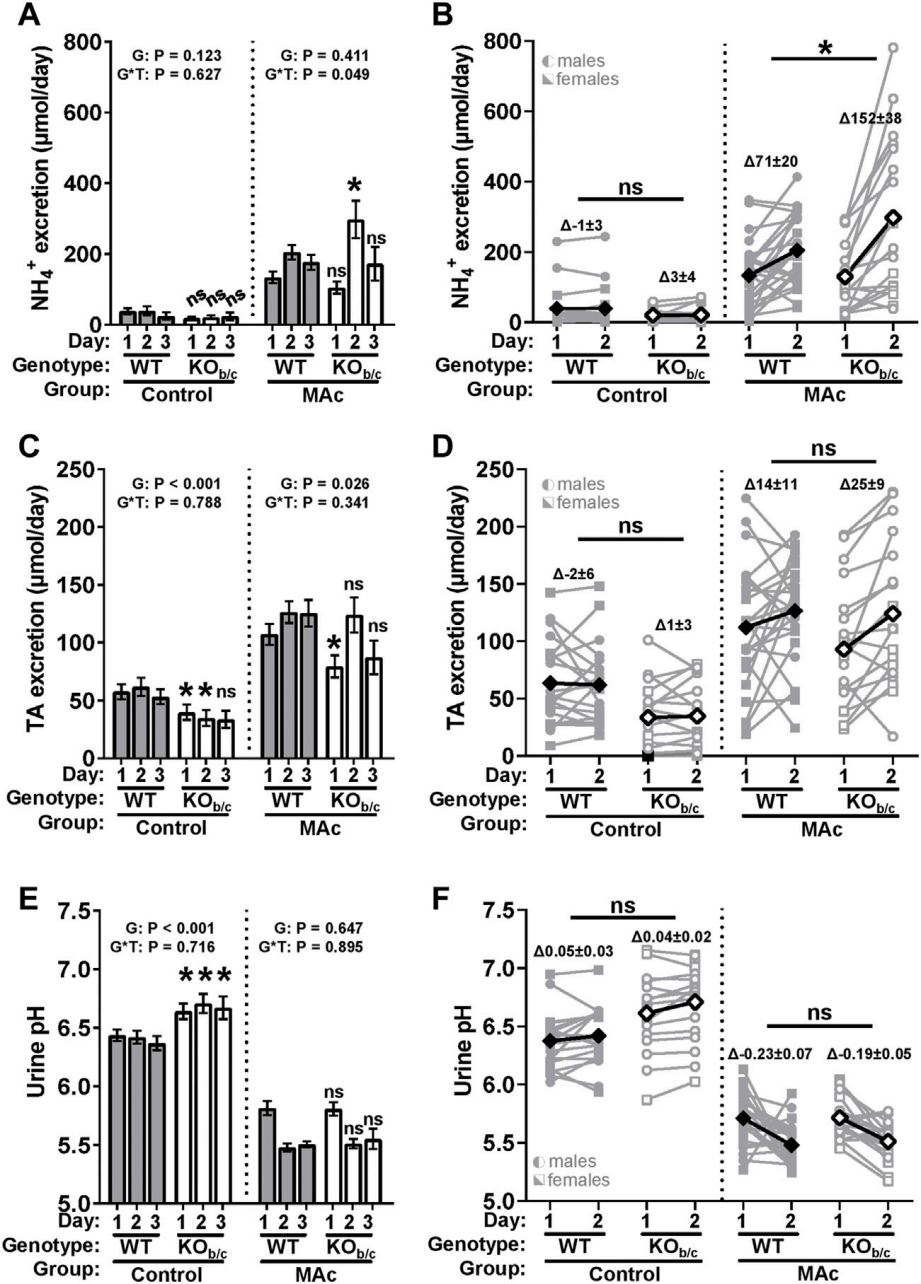
KO_b/c_ renal acid-excretion during 3-day MAc-challenge. 24-h urine collections were obtained for each day of a 3-day MAc-challenge (timepoints “1–3”) and used to assess daily NH_4_^+^ excretion **(A,B)**, titratable acid (TA) excretion **(C,D)**, and urine pH **(E,F)**. **(A)** Daily NH_4_^+^ excretion. Under control conditions there was no significant difference in average daily NH_4_^+^ excretion between WT and KO_b/c_ mice. Under MAc conditions, there was a significant genotype × time interaction effect (*p* = 0.049, ANOVA), with KO_b/c_ mice having excreted significantly more NH_4_^+^ than WT mice on day 2 of the MAc-challenge. **(B)** Change in NH_4_^+^ excretion between day 1 and 2 (ΔNH_4_^+^ day_1-2_). There was no significant difference between WT and KO_b/c_ ΔNH_4_^+^ day_1-2_ under control conditions. Under MAc conditions, ΔNH_4_^+^ day_1-2_ in KO_b/c_ mice was significantly greater than in WT mice. **(C)** Daily TA excretion. Under control and MAc conditions, daily average TA excretion was significantly less in KO_b/c_ mice than in WT mice. **(D)** Change in TA excretion between day 1 and 2 (ΔTA day_1-2_). There was no significant difference between WT and KO_b/c_ ΔTA day_1-2_ under control or MAc conditions. **(E)** Daily urine pH. Under control conditions, the average daily urine pH was significantly greater in KO_b/c_ mice than in WT mice, but under MAc conditions, there was no significant difference in daily average urine pH. **(F)** Change in urine pH between day 1 and 2 (Δurine pH day_1-2_). There was no significant difference between WT and KO_b/c_ Δurine pH day_1-2_ under control or MAc conditions. *p*-values reported for the main effect of genotype (“G”) and genotype x time (“G*T”) interaction effect in A, C, and E. Diamond symbols in B, D, and F represent average values. *p < 0.05 between WT and KO_b/c_ mice at each day noted, assessed by Student’s 2-tailed unpaired *t*-test or Mann-Whitney test; ns, non-significant. For n’s in panels A, C, and E, see Table 1. For B, D, and F – [control] WT: n = 11M/9F, KO_b/c_: n = 11M/7F-[MAc] WT: n = 11M/13F, KO_b/c_ n = 11M/8F.

Under control conditions, there was no significant difference between WT and KO_b/c_ daily average NH_4_^+^ excretion (Figure 7A, control). Under MAc-conditions, there was a significant genotype x time (G*T) interaction effect (*p* = 0.049 ANOVA), indicating a possible difference in the time course of WT and KO_b/c_ NH_4_^+^ excretion. Indeed, on day 2 of MAc, KO_b/c_ mice excreted significantly more NH_4_^+^ than WT mice (*p* = 0.018; Figure 7A, MAc, timepoint 2). To determine if this peak in KO_b/c_ NH_4_^+^ excretion over day 2 was a result of increased NH_4_^+^ excretion in individual mice, we calculated the change in NH_4_^+^ excretion that occurred in each animal between days 1 and 2 (ΔNH_4_^+^ day_1-2_, Figure 7B). Under control conditions there was no significant difference in ΔNH_4_^+^ day_1-2_ (Figure 7B, control). However, under MAc conditions, KO_b/c_ mice had a significantly greater ΔNH_4_^+^ day_1-2_ than WT mice (*p* = 0.046; Figure 7B, MAc).

Along with increased NH_4_^+^ excretion, increased TA excretion and urine acidification are also expected during MAc; thus, we similarly assessed TA excretion and urine pH in WT and KO_b/c_ mice (Figures 7C–F). Under control and MAc conditions, the daily average TA excretion was significantly less in KO_b/c_ mice than WT mice (G: *p* < 0.001 and *p* = 0.026; Figure 7C). However, the change in TA excretion between days 1 and 2 (ΔTA day_1-2_) was not significantly different between WT and KO_b/c_ mice under control conditions or MAc-conditions (Figure 7D, control and MAc). Average daily urine pH was significantly greater in KO_b/c_ mice than WT mice while under control conditions (G: *p* < 0.001; Figure 7E, control). However, during MAc, there was no significant difference between the average urine pH of WT and KO_b/c_ mice. Lastly, the change in urine pH between days 1 and 2 (Δurine pH day_1-2_) was not significantly different between WT and KO_b/c_ mice under control conditions or MAc-conditions (Figure 7F, control and MAc).

Table 2 displays relevant metabolic and electrolyte daily averages measured in WT and KO_b/c_ mice during the 1–3-days of control (0.5% sucrose added to drinking water) or MAc (0.28M NH_4_Cl + 0.5% sucrose added to drinking water) challenged conditions. To summarize these results, KO_b/c_ mice were significantly smaller by body-weight, and ate and drank less compared to their WT counterparts. However, when food and fluid intake were normalized to bodyweight these differences in intake became non-significant. Under control conditions, KO_b/c_ mice had a significantly lower [Na^+^] [Cl^−^], and BUN. During MAc [Cl^−^] and BUN both remained significantly lower in KO_b/c_ mice compared to WT, while there was no significant difference in [Na^+^]. Overall, these results make it unlikely that differences in fluid intake, food intake, hydration status, or metabolism of NH_4_Cl, account for the observed differences in renal acid-excretion.

Since both male and female mice were used in these experiments, we assessed for differences in all parameters by including sex as an independent factor in ANOVA. Notably, only NH_4_^+^ excretion under the MAc condition was found to have a significant sex interaction (genotype x time x sex: *p* = 0.047). Analysis of the sex-split data set suggests that male KO_b/c_ mice are the primary drivers of the increase in NH_4_^+^ excretion observed in KO_b/c_ mice on day 2 of MAc (Day 2 [males]–WT: 216 ± 30 µmol/day, KO_b/c_: 411 ± 65 µmol/day, *p* = 0.001, n = 11 per group; Day 2 [females]–WT: 157 ± 22 µmol/day, KO_b/c_: 120 ± 29 µmol/day, *p* = 0.460, n = 13 and 7, respectively). Possible explanations for this sex difference are discussed below (see ‘Discussion’ section); however, since characterization of sex-specific differences was not a primary aim of this study and we did not observe any significant sex-interactions in any other parameters, data from male and female mice were kept pooled for reporting in Figure 7. Altogether, these data suggest that NH_4_^+^ excretion, but not TA excretion or urine acidification, is enhanced in KO_b/c_ mice over day 2 of MAc.

### 3.7 Effect of NBCe1-B loss on ammonia metabolism

**FIGURE 8:**
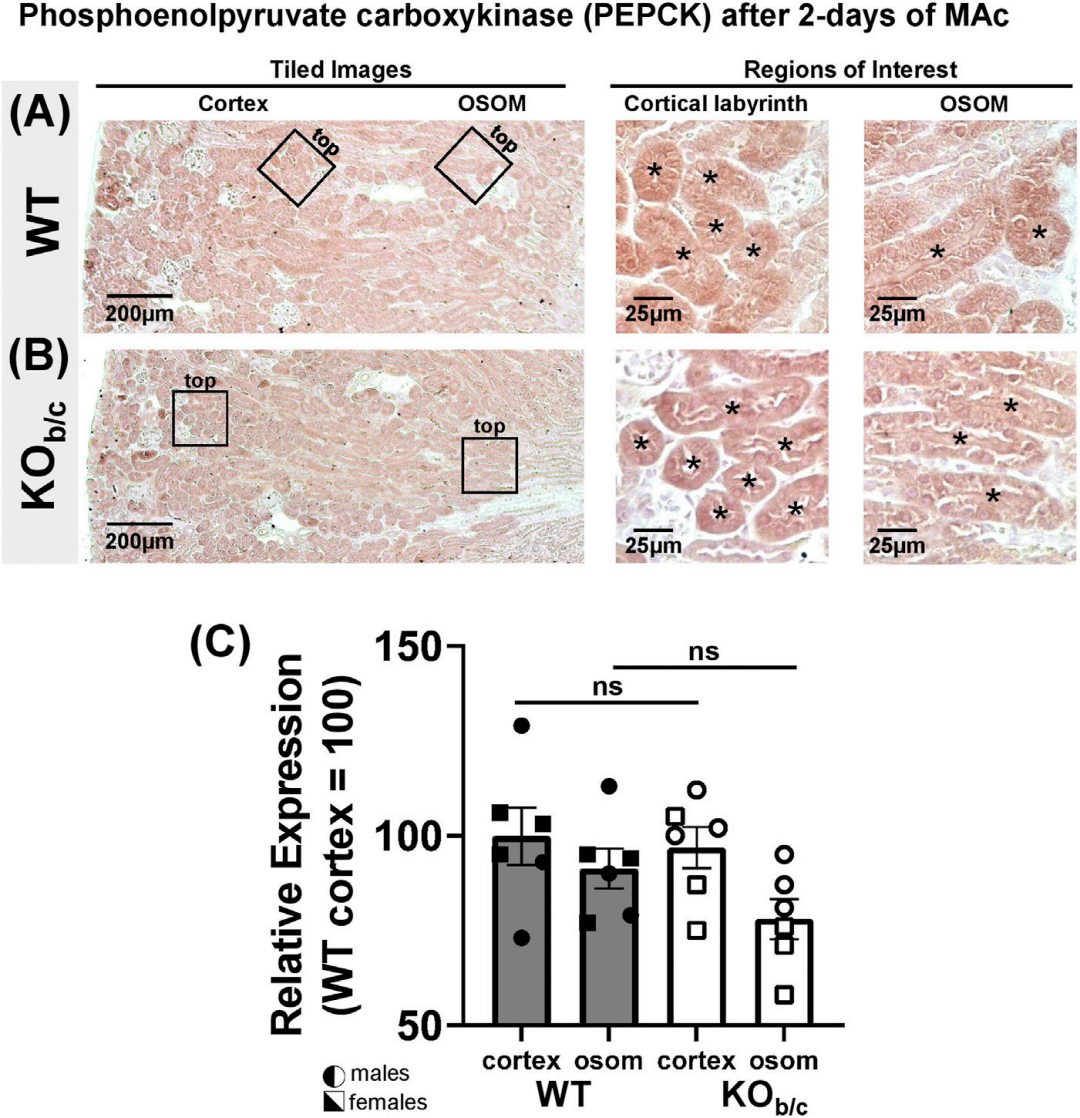
Phosphoenolpyruvate carboxykinase (PEPCK) kidney expression in WT and KO_b/c_ mice after 2-day MAc-challenge. **(A,B)** Representative low and high magnification light-microscopy images of PEPCK immunostaining in WT **(A)** and KO_b/c_ **(B)** mice exposed to 2-days of MAc-challenged conditions. High magnification images of cortex and OSOM regions of interest were used for quantification of PEPCK expression in each region. Asterisks signify tubule lumens. **(C)** Quantification of PEPCK expression in the cortex and OSOM of WT and KO_b/c_ mice normalized such that the average in WT cortex is 100%. Comparisons between WT and KO_b/c_ groups assessed by Students 2-tailed unpaired *t*-test; ns, non-significant. N = 3M/3F per group.

**FIGURE 9:**
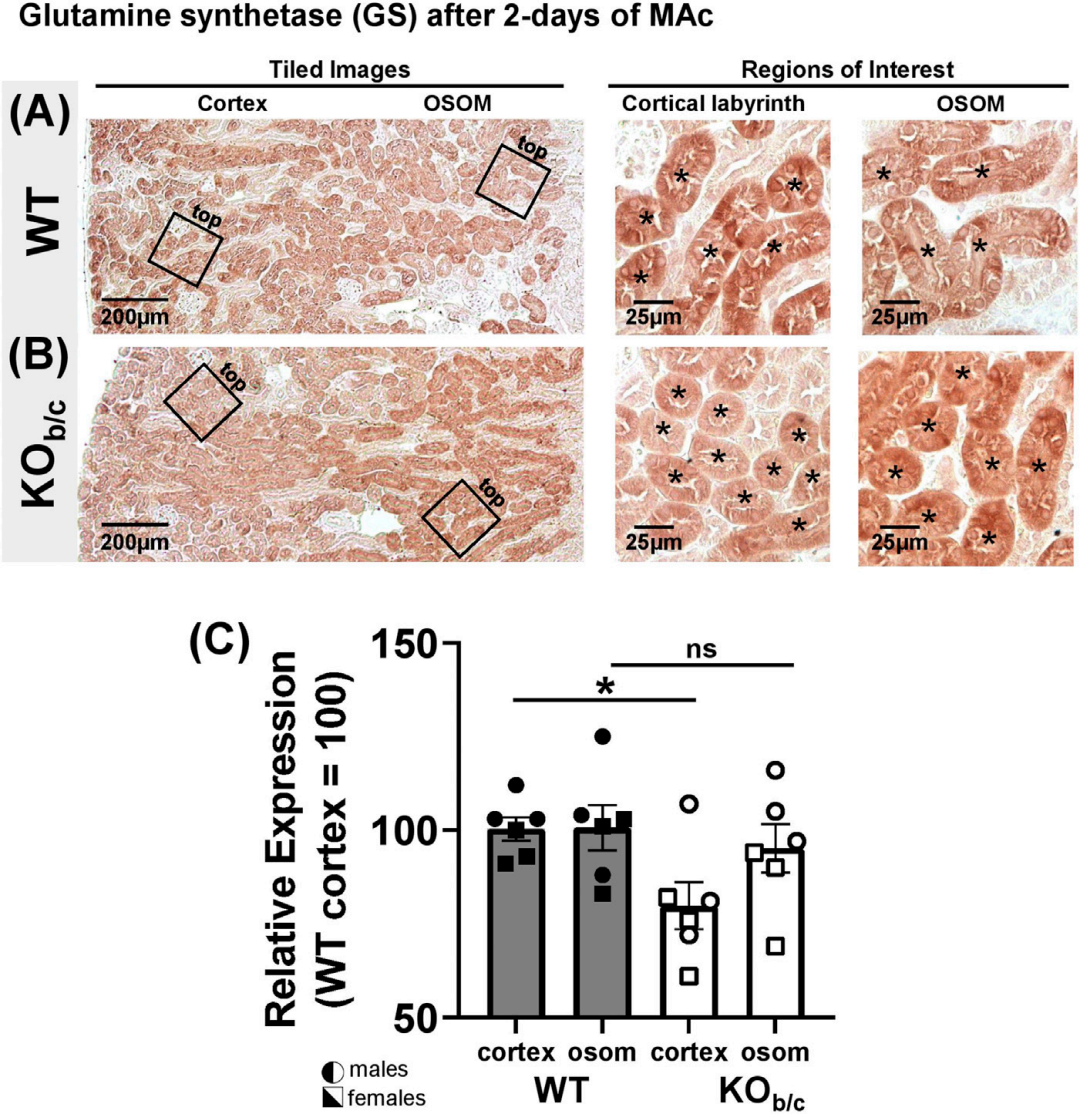
Glutamine synthetase (GS) kidney expression in WT and KO_b/c_ mice after 2-day MAc-challenge. **(A,B)** Representative low and high magnification light-microscopy images of GS immunostaining in WT **(A)** and KO_b/c_ **(B)** mice exposed to 2-days of MAc-challenged conditions. High magnification images of cortex and OSOM regions of interest were used for quantification of GS expression in each region. Asterisks signify tubule lumens. **(C)** Quantification of GS expression in the cortex and OSOM of WT and KO_b/c_ mice normalized such that the average in WT cortex is 100%. Comparisons between WT and KO_b/c_ groups assessed by Students 2-tailed unpaired *t*-test; *p < 0.05; ns, non-significant. N = 3M/3F per group.

Results of recent studies in NBCe1-A KO mice and combined NBCe1-A/B kidney specific KO mice have indicated that both NBCe1-A and NBCe1-B are important for the normal upregulation of ammoniagenesis during MAc (Lee et al., 2018; 2022). Thus, we were surprised to observe an increase (rather than decrease) in NH_4_^+^ excretion in KO_b/c_ mice during MAc (Figure 7). To this end, we hypothesized that in KO_b/c_ mice, ammoniagenesis is enhanced in PTs of the cortical labyrinth, where only NBCe1-A and not NBCe1-B is expressed (Figure 3). To test this hypothesis, we compared the expression of PEPCK and GS between WT and KO_b/c_ PTs located in the cortical labyrinth and OSOM after 2-days of MAc-challenged conditions. MAc normally stimulates an increase in PEPCK expression and a decrease in GS expression. We found no significant difference between WT and KO_b/c_ PEPCK expression in the cortex or in the OSOM (cortex, *p* = 0.743; OSOM, *p* = 0.112; Figures 8A–C), indicating PEPCK expression is stimulated to a similar extent in WT and KO_b/c_ mice. Conversely, GS expression in the cortex was significantly lower in KO_b/c_ mice than WT mice (*p* = 0.016), while there was no significant difference between WT and KO_b/c_ GS expression in the OSOM (*p* = 0.550; Figures 9A–C). This result indicates that the decrease in GS expression expected during MAc is enhanced in PTs of the cortical labyrinth of KO_b/c_ mice, overall supporting the hypothesis that ammoniagenesis is stimulated to a greater extent in KO_b/c_ mice.

## 4 Discussion

This study provides novel information regarding the molecular mechanisms underlying the renal and respiratory responses to acidosis. In summary: we investigated NBCe1-B expression in the WT kidney, finding NBCe1-B to be expressed in PTs of the cortical medullary ray and OSOM, but not in PTs of the cortical labyrinth (where NBCe1-A is specifically expressed). Moreover, in response to MAc, NBCe1-B abundance significantly increased in the WT kidney, while there was no change in NBCe1-A abundance in either WT or KO_b/c_ kidneys. We further demonstrate the absence of NBCe1-B/C expression in the brainstem of KO_b/c_ mice. Consequently, during MAc, KO_b/c_ mice do not exhibit the expected increase in minute ventilation and have a more severe decrease in plasma pH than WT mice as they are unable to blow off CO_2_. Surprisingly, KO_b/c_ mice ultimately recovered their plasma pH to the same level as WT mice, suggesting that the renal response is compensating for the impaired respiratory response during MAc, even in the absence of renal NBCe1-B. Indeed, we observed enhanced urinary NH_4_^+^ excretion in KO_b/c_ mice during MAc; a result that is seemingly at odds with results of recent studies indicating that loss of NBCe1-B, at least in the context of the severe acidosis imposed by concurrent NBCe1-A loss, further impairs ammoniagenesis during MAc (Lee et al., 2022). To this end, in the kidneys of KO_b/c_ mice we observed significantly less GS expression in PTs located in the cortical labyrinth, supporting our hypothesis that renal ammoniagenesis is enhanced in KO_b/c_ mice during MAc.

Historically, NBCe1-A was considered the only renal NBCe1 variant. However, it was recently discovered that NBCe1-B is also expressed in the kidney, albeit at lower abundance than NBCe1-A (Fang et al., 2018). Specifically, NBCe1-B kidney expression was determined in NBCe1-A KO mice (Romero et al., 2014; Lee et al., 2018); with total-NBCe1-immunoreactivity (representing NBCe1-B in the context of NBCe1-A KO) demonstrated in some PTs of the cortical labyrinth, and more robustly in PTs located in both the medullary ray in the cortex and the OSOM (Fang et al., 2018). However, because NBCe1-B transcripts are expressed from an acid-induced promoter (Snead et al., 2011) and because NBCe1-A KO mice are congenitally acidemic, it is unclear whether the renal expression of NBCe1-B in NBCe1-A KO mice is representative of that in WT mice. Therefore, using a novel, commercially available, NBCe1-B/C specific antibody, here we demonstrate positive NBCe1-B immunoreactivity in WT kidney PTs located in the cortical medullary ray and OSOM. However, we did not observe NBCe1-B immunoreactivity in PTs of the cortical labyrinth. Thus, NBCe1-B expression in PTs of the cortical labyrinth may be a unique feature of the NBCe1-A specific KO mouse, attributable to the severe and chronic spontaneous MAc characteristic of NBCe1-A KO mice. Nonetheless, in-line with previous reports (Fang et al., 2018) we observed a significant increase in renal NBCe1-B expression during MAc, while no such acid-sensitivity was observed for NBCe1-A expression. Importantly, the lack of increased NBCe1-A expression does not preclude the possibility that MAc stimulated a per molecule increase in NBCe1-A activity in PTs of KO_b/c_ mice. It is well established that NBCe1-A activity increases in response to MAc without a change in abundance [reviewed in (Parker and Boron, 2013)]; thus, since our evidence suggests an enhanced ammoniagenic response in KO_b/c_ PTs of the cortical labyrinth, PTs where NBCe1-A is expressed, this supports the hypothesis MAc stimulates NBCe1-A activity through a post-translational mechanism (Alsufayan et al., 2021).

The second key finding of this study is the lack of a respiratory response and elevated pCO_2_ in KO_b/c_ mice during MAc, which supports *in vitro* data suggesting that an NBCe1-B/C mediated mechanism underlies the chemosensitivity of the RTN (Turovsky et al., 2016). While the molecular mechanisms underlying the chemosensitivity of the RTN are still controversial, one hypothesis posits that a decrease in pH_i_ of astrocytes activates NBCe1-B/C and the influx of Na^+^ reverses Na^+^/Ca^2+^ exchange (NCX), triggering Ca^2+^-dependent ATP release (Turovsky et al., 2016). ATP then activates adjacent RTN neurons through P2Y-receptor purinergic signaling (Gourine et al., 2010; Turovsky et al., 2016; Guyenet et al., 2019). In line with this hypothesis, here we demonstrate that KO_b/c_ mice do not exhibit the same increase in minute volume or tidal volume in response to MAc as WT mice, which to our knowledge is the first *in vivo* report of an impaired respiratory response to MAc attributable to NBCe1-B/C loss. We cannot discount the possibility that CO_2_ responsiveness is disturbed at other chemosensitive sites in KO_b/c_ mice, contributing to the observed phenotype. Little is known about the molecular mechanisms of pH regulation in these regions, although pH regulation in chemosensitive neurons of the medullary raphe and locus coeruleus is linked to acid extrusion mediated by Na^+^/H^+^ exchangers or bicarbonate transporters other than NBCe1 (Kersh et al., 2009; Coley et al., 2013).

Although minute volume did not significantly change from baseline in KO_b/c_ mice, a significant increase in tidal volume was still observed (albeit significantly less than the WT tidal volume response), which suggests the ventilatory response to MAc is partially intact. This may be a result of other chemosensitive nuclei within the central respiratory network, as well as from contributions of peripheral chemoreceptors such as the aortic and carotid bodies (Guyenet and Bayliss, 2015). We also observed a decrease in frequency in both WT and KO_b/c_ mice during MAc that was unexpected since an increase in both tidal volume and frequency might be expected to underlie an increase in minute volume. This could partially be explained by the fact that metabolic inputs, such as acidemia, preferentially affect tidal volume, as has been shown in both human and animal studies (Borison et al., 1977; Javaheri et al., 1982; Nicolò et al., 2017; 2018; Tipton et al., 2017). Lastly, we observed a small, but significant, difference in baseline minute volume, with KO_b/c_ mice exhibiting a higher minute volume, which coincided with a significantly lower baseline pCO_2_. At this time, we cannot explain this baseline difference in ventilation, although we note that NBCe1-B/C is broadly expressed in histologic sections from the brainstem medulla of WT mice (Figure 2) and thus may contribute to regions beyond the RTN, including those involved with controlling baseline ventilation.

**FIGURE 10:**
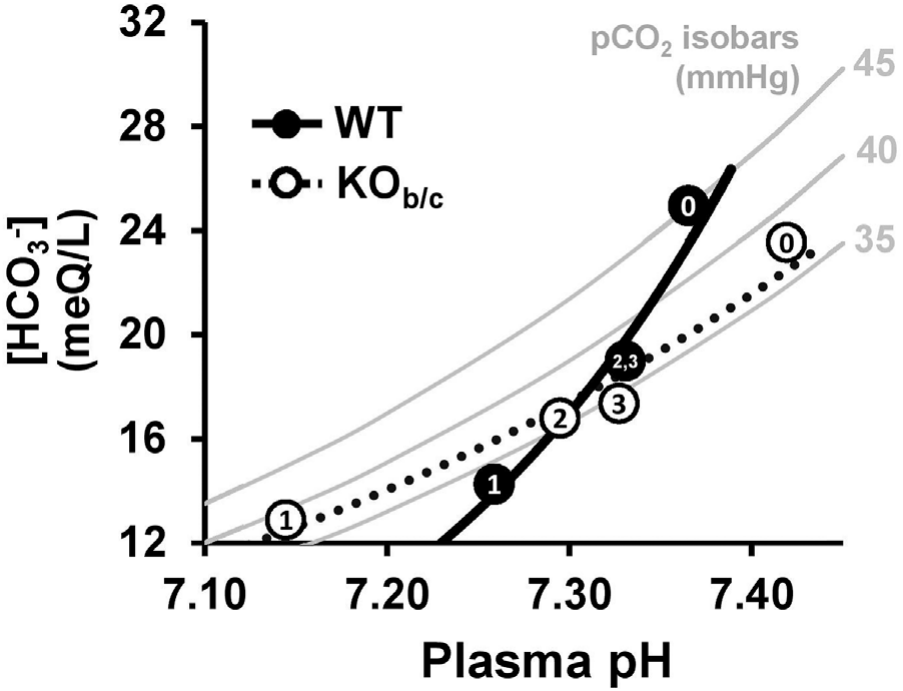
Davenport diagram illustrating acid-base status of WT and KO_b/c_ mice during 3-day MAc-challenge. Grey bars represent pCO_2_ isobars for pCO_2_ levels of 35, 40, and 45 mmHg, which represent all the possible combinations of plasma pH and [HCO_3_^−^] for the given pCO_2_ level according to the Henderson-Hasselbalch relationship. The average of each acid-base parameter at baseline and during each day of the 3-day MAc-challenge are represented as filled circles for WT mice and open circles for KO_b/c_ mice, with the numbers (0–3) representing each day of the experiment (data taken from Figure 6). Both data sets were fitted with an exponential trend line. Note the crossing of all 3 isobars by the WT trend line whereas the KO_b/c_ trend line closely follows the 35 mmHg isobar. This reflects the intact ability of WT mice to adjust respiration given the severity of acidosis whereas in KO_b/c_ mice this respiratory response is impaired.

Despite these baseline differences, our data indicate that the impaired respiratory response to MAc underlies the more severe acidemia observed in KO_b/c_ mice after the first day of MAc-challenged conditions (Figure 6E, timepoint 1). Surprisingly however, after 2 and 3 days of MAc-challenged conditions, the plasma pH of KO_b/c_ mice was not significantly different from WT, which suggests compensation by the kidney in defense of overall plasma pH. Additionally, the recovery of plasma pH in both WT and KO_b/c_ mice likely explains the pattern of ventilation observed over the 3-day MAc-challenge; that is since the respiratory response is proportional to the severity of acidemia, as pH recovers the magnitude of the ventilatory response returns towards baseline. This integrated response is more easily described through the use of a Davenport diagram in which all three acid-base parameters (pH, pCO_2_, and HCO_3_^−^) can be displayed (Figure 10). The grey bars in Figure 10 represent the pCO_2_ isobars for pCO_2_ levels of 35, 40, and 45 mmHg, respectively, which illustrate all the possible combinations of plasma pH and [HCO_3_^−^] for the given pCO_2_ level according to the Henderson-Hasselbalch relationship. By adding in the measured acid-base parameters from WT (filled circles) and KO_b/c_ (open circles) mice during the 3-day MAc-challenge (taken from Figure 6; numbers in data points represent experimental timepoints) and fitting exponential trendlines to these data, one can appreciate that the WT trendline crosses all three isobars. This represents the intact ability of WT mice to adjust their pCO_2_ based on the severity of acidosis via the respiratory response to MAc. In contrast, the KO_b/c_ trendline closely follows the 35 mmHg pCO_2_ isobar, indicative of an impaired respiratory response in KO_b/c_ mice during MAc. Nonetheless, the ability to recover pH remains largely intact in KO_b/c_ mice, which we attribute to the production and absorption of additional HCO_3_^−^ by the kidney.

Kidney ammoniagenesis accounts for the majority of HCO_3_^−^ production during MAc (Weiner and Verlander, 2019), and indeed KO_b/c_ mice exhibited enhanced NH_4_^+^ excretion during the MAc-challenge (Figure 7B). Considering that the respiratory response to MAc occurs on the order of seconds to minutes, while the kidney response takes hours to days to develop (Adrogué and Madias, 2010), we believe this enhancement of NH_4_^+^ excretion likely represents renal compensation for the impaired respiratory response. The link between the impaired respiratory response and the enhanced renal response appears to be pH; that is, after the first day of MAc, KO_b/c_ mice exhibited a more severe acidemia than WT mice (Figure 6E, timepoint 1), which likely stimulated renal NH_4_^+^ excretion over day 2 (Figures 7A, B). Once KO_b/c_ plasma pH recovered to the same level as WT, which occurred by the end of day 2, the ammoniagenic requirement was only to match the daily consumption of HCO_3_^−^ due to the daily intake of NH_4_Cl, which likely explains why we observed a return to WT levels of NH_4_^+^ excretion in KO_b/c_ mice on day 3. Finally, we cannot completely discount the possibility that the lack of a decrease in pCO_2_ from baseline in KO_b/c_ mice contributed to the enhancement of renal ammoniagenesis independently from pH, since CO_2_ alone has been demonstrated to stimulate renal acid excretion and PT HCO_3_^−^ reabsorption (Madias et al., 1977; Zhao et al., 2003; Zhou et al., 2005).

There were no significant differences in overall TA excretion or urinary acidification between genotypes during the 3-day MAc-challenge, indicating urinary acidification mechanisms are intact in KO_b/c_ mice. However, under control conditions KO_b/c_ mice exhibited significantly lower TA excretion and significantly higher urinary pH. TA excretion partially depends on urinary pH (Mioni and Mioni, 2014). Hence, lower TA excretion may be a symptom of the more alkaline urine pH. The elevation in urine pH could be indicative of HCO_3_^−^ wasting in KO_b/c_ mice at baseline; however, this is likely to be a mild occurrence as we observed no significant difference in baseline plasma [HCO_3_^−^]. An explanation for these seemingly contradictory findings is that the small quantity of urinary HCO_3_^−^ necessary to account for the observed difference in urine pH, likely would not cause a detectable difference in plasma [HCO_3_^−^]. We also note the presence of an apparent sex-difference in NH_4_^+^ excretion, in which the enhanced NH_4_^+^ excretion during MAc appears to be driven by KO_b/c_ males. No other sex-differences were observed throughout the study, and we currently do not have an explanation for this potential sex-difference in NH_4_^+^ excretion. Although, previous studies have demonstrated sex-differences in ammoniagenesis. Specifically, in response to MAc, NH_4_^+^ excretion increased to a greater extent in WT males than females (Harris et al., 2018; 2019). Furthermore, in male mice, PT size and density in the cortex is greater than in females; whereas in female mice, intercalated cells are larger and there is a higher density of collecting ducts than in males (Harris et al., 2019). Together, these anatomical differences support the possibility of a greater ammoniagenic response in males.

Irrespective of the potential sex-difference, we were surprised to observe increased NH_4_^+^ excretion in KO_b/c_ mice compared to WT mice (rather than decreased), since several recent findings by others have suggested a role for NBCe1-B in ammoniagenesis (Fang et al., 2018; Lee et al., 2018; 2022). Moreover, the data presented here demonstrating that NBCe1-B abundance increases during MAc (Figure 4) further supports a role for NBCe1-B in the renal response to MAc. Nevertheless, it appears the stimulatory condition imposed by the impaired respiratory response of KO_b/c_ mice overshadows any defect in ammoniagenesis due to NBCe1-B loss. We believe this is largely attributable to enhancement of ammoniagenesis in PTs located in the cortical labyrinth; PTs in which NBCe1-A is specifically expressed (Schmitt et al., 1999; Maunsbach et al., 2000). This hypothesis is supported by our observations of no difference in PEPCK expression and significantly less GS expression in KO_b/c_ *versus* WT cortical PTs. PEPCK is involved with catalyzing the formation of HCO_3_^−^ from α-ketoglutarate (the end-product of glutamine deamination) such that MAc normally stimulates increased PEPCK expression (Curthoys and Gstraunthaler, 2001). GS is involved with the recycling of NH_4_^+^ by catalyzing the formation of glutamine from glutamate, hence MAc usually stimulates a decrease in GS expression (Conjard et al., 2003). Therefore, the lower expression of GS in PTs of the KO_b/c_ renal cortex supports our hypothesis that ammoniagenesis is enhanced in the NBCe1-A expressing cortical PTs of KO_b/c_ mice, likely overshadowing any impairment in OSOM ammoniagenesis resulting from the absence of NBCe1-B. Nevertheless, we cannot entirely discount the possibility that stimulation of other components of renal ammonium handling, such as the Rhesus glycoproteins, Rh B and Rh C Glycoproteins (Bishop et al., 2010; Weiner and Verlander, 2014), could contribute to the observed enhancement of NH_4_^+^ excretion.

**FIGURE 11:**
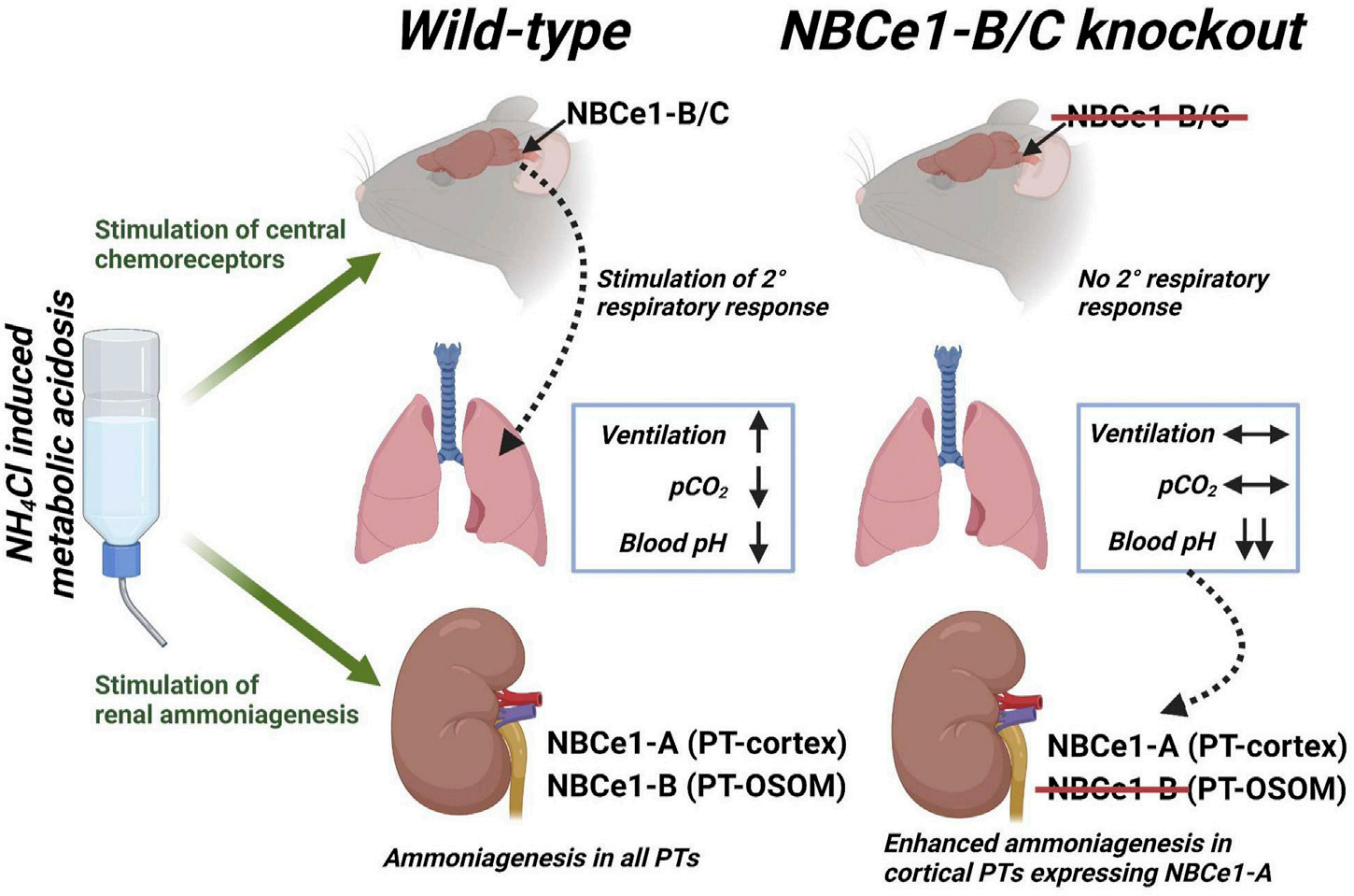
Illustration of the effect of NBCe1-B/C absence on the integrated physiologic response to MAc. Induction of MAc by NH_4_Cl stimulates central chemoreceptors that trigger the secondary respiratory response. The resulting increase in ventilation lowers pCO_2_, which helps mitigate the primary fall in plasma pH. Concurrently, MAc stimulates renal ammoniagenesis in attempt to increase HCO_3_^−^ production to replace the HCO_3_^−^ consumed in buffering the acid-load. In the absence of NBCe1-B/C, as demonstrated by KO_b/c_ mice, the secondary respiratory response to MAc is impaired. Therefore, there is no change in pCO_2_ during MAc, which leads to an initial greater severity of acidemia. However, NBCe1-A expression in the kidney remains intact, specifically in proximal tubules (PTs) located in the cortical labyrinth. Therefore, the greater severity of acidemia in KO_b/c_ mice prompts an enhancement of ammoniagenesis in NBCe1-A expressing PTs that ultimately recovers plasma pH equal to that of wild-type mice. This enhancement of ammoniagenesis appears to overshadow any potential defect in ammoniagenesis resulting from NBCe1-B loss (NBCe1-B being usually expressed in PTs of the outer segment of the outer medulla (OSOM)).

The proposed effect of NBCe1-B/C absence on the integrated physiologic response to MAc is illustrated in Figure 11. In summary, these investigations of the KO_b/c_ response to MAc adds to the growing body of literature regarding the role of NBCe1 variants in acid-base physiology. Here we found that NBCe1-B is expressed in the WT kidney at baseline and increases in response to MAc, supporting the hypothesis that NBCe1-B contributes to renal regulation of plasma pH. However, the global loss of NBCe1-B in KO_b/c_ mice, with the resulting impaired respiratory response to MAc, precludes any definitive conclusion as to the role of kidney NBCe1-B during acidosis. Nevertheless, this study provides critical new insight into the necessary role of NBCe1-B/C in the respiratory response to MAc, and demonstrates the complexity of the mechanisms regulating acid-base homeostasis.

## Data Availability

The raw data supporting the conclusion of this article will be made available by the authors, without undue reservation.
